# p53 positively regulates the proliferation of hepatic progenitor cells promoted by laminin-521

**DOI:** 10.1038/s41392-022-01107-7

**Published:** 2022-08-31

**Authors:** Mingyang Ma, Shuyao Hua, Xiangde Min, Liang Wang, Jun Li, Ping Wu, Huifang Liang, Bixiang Zhang, Xiaoping Chen, Shuai Xiang

**Affiliations:** 1 Department of General Surgery, Tianjin Medical University General Hospital, Tianjin, China; 2 Hepatic Surgery Center, Tongji Hospital, Tongji Medical College, Huazhong University of Science and Technology, Wuhan, China; 3 Department of Clinical Nutrition, Shanghai Jiao Tong University Affiliated Sixth People’s Hospital, Shanghai, China; 4 Department of Radiology, Tongji Hospital, Tongji Medical College, Huazhong University of Science and Technology, Wuhan, China; 5 Wuhan Pulmonary Hospital, Wuhan Institute for Tuberculosis Control, Wuhan, China; 6 Department of Pathophysiology, Tongji Medical College, Huazhong University of Science and Technology, Wuhan, China; 7 Hubei Key Laboratory of Hepato-Pancreato-Biliary Disease, Wuhan, China; 8 Key Laboratory of Organ Transplantation, Ministry of Education, Wuhan, China; 9 Key Laboratory of Organ Transplantation, National Health Commission, Wuhan, China; 10 Key Laboratory of Organ Transplantation, Chinese Academy of Medical Sciences, Wuhan, China

**Keywords:** Adult stem cells, Stem-cell research

## Abstract

Hepatic progenitor cells (HPCs) hold tremendous potential for liver regeneration, but their well-known limitation of proliferation hampers their broader use. There is evidence that laminin is required for the proliferation of HPCs, but the laminin isoform that plays the dominant role and the key intracellular downstream targets that mediate the regulation of HPC proliferation have yet to be determined. Here we showed that p53 expression increased gradually and reached maximal levels around 8 days when laminin α4, α5, β2, β1, and γ1 subunit levels also reached a maximum during HPC activation and expansion. Laminin-521 (LN-521) promoted greater proliferation of HPCs than do laminin, matrigel or other laminin isoforms. Inactivation of p53 by PFT-α or Ad-p53^V143A^ inhibited the promotion of proliferation by LN-521. Further complementary MRI and bioluminescence imaging analysis showed that p53 inactivation decreased the proliferation of transplanted HPCs in vivo. p53 was activated by LN-521 through the Integrin α6β1/FAK-Src-Paxillin/Akt axis. Activated p53 was involved in the nuclear translocation of CDK4 and inactivation of Rb by inducing p27^Kip1^. Taken together, this study identifies LN-521 as an ideal candidate substrate for HPC culture and uncovers an unexpected positive role for p53 in regulating proliferation of HPCs, which makes it a potential target for HPC-based regenerative medicine.

## Introduction

Hepatic progenitor cell therapy holds tremendous potential for the treatment of end-stage liver disease. Nevertheless, numerous hurdles remain to be overcome before it can be effectively used in clinical practice. A major challenge is the large-scale expansion of HPCs, which are usually quiescent and rarely detected in normal adult livers, instead only proliferating in cases of severely impaired hepatocyte proliferation.^1^ Therefore, it is critically important to study efficient approaches for the regulation of HPC proliferation with the underlying molecular mechanisms. Laminin is one of the major extracellular matrix (ECM) proteins of the liver, and is closely associated with proliferation and differentiation of HPCs during liver development.^2^ In CCl_4_ or CDE induced chronic liver injury, dense laminin deposits appear before HPC activation in vivo.^3^ In vitro studies further showed that laminin can support the proliferation of HPCs in an undifferentiated form.^4,5^ However, other studies found that laminin induced the differentiation of cultured HPCs into cholangiocytes.^6^ This discrepancy might be caused by the differences between laminin isoforms formed by various combinations of α, β and γ chains.^7^ Based on this premise, we examined the variation of laminin subunits in the process of HPC activation and proliferation induced by 2AAF/PH. Furthermore, we also assessed the effects of different laminin isoforms on HPC proliferation in comparison with other major ECM proteins, and identified LN-521 as having the greatest effect on promoting the proliferation of HPCs in vitro.

The well-known tumor suppressor p53 was traditionally considered to induce cell cycle arrest and apoptosis.^8^ However, recent studies identified a contribution of p53 to cell survival and proliferation. In proliferating hepatocytes, p53 has been shown to activate downstream signals that maintain hepatocyte proliferation.^9^ A high level of p53 protein was found in the cytoplasm of mouse embryonic stem cells, where it contributes to the maintenance of self-renewal and induction of proliferation.^10^ Interestingly, we also found high hepatic p53 protein levels in rats following 2-AAF administration and partial hepatectomy spanning two thirds of the liver. As HPCs extensively expand to compensate the loss of liver mass during this process, it is possible that p53 acts as a positive regulator that initiates HPC proliferation. Moreover, the upregulation of p53 was accompanied by the upregulation of laminin subunits α5, β2 and γ1, so we hypothesized that p53 may contribute to HPC proliferation by interacting with LN-521. Further investigation is required to clarify whether and how p53 interacts with LN-521 to induce HPC proliferation.

The protein level of p53 is determined by both transcriptional regulation and posttranslational modification, whereby the latter is closely regulated by the PI3K/Akt-mediated ubiquitin proteasome pathway.^11^ It has been reported that the accumulation of p53 induced by DNA damage in ﻿mouse embryonic fibroblasts is prevented by PI3K inhibition.^12^ By contrast, the expression of constitutively active Akt in MCF-7 breast cancer cells diminished the cellular levels of p53.^13^ MEK/ERK signaling was also shown to control p53 levels.^14^ ﻿For example, the activation of MEK/ERK is required for hematopoietic growth factor-induced neurite outgrowth by upregulating the levels of p53 protein.^15^ However, inhibition of the MEK/ERK pathway by U0126 or PD98059, but not PI3K signaling, was shown to prevent ﻿transforming﻿ growth factor-induced p53 degradation in hepatocytes.^16^ The conflicting data on the effects of PI3K/Akt or MEK/ERK on p53 may be attributed to differences of stimuli and cell types. Although the mechanisms responsible for the upregulation of p53 protein levels in proliferating HPCs are still unclear, we provide evidence for the involvement of Akt-dependent and ERK-independent signaling in the LN-521 induced accumulation of p53 in proliferating HPCs.

The p21^Cip1^ and p27^Kip1^ proteins, which can selectively bind to and inhibit the activity of cyclin-cyclin-dependent kinase (CDK) complexes, are two of the best characterized cell proliferation regulators downstream of p53.^17,18^ However, the fact that they can be found in complexes with active cyclin-CDKs challenges this widely prevailing view, and there is increasing evidence that beyond their roles as CDK inhibitors, p21^Cip1^ and p27^Kip1^ could potentially function as activators of these kinases.^19–21^ In proliferating hepatocytes, expression of p21^Cip1^ protein was increased in response to EGF stimulation,^22^ and this specific upregulation of p21^Cip1^ was further shown to induce CDK2 phosphorylation and nuclear accumulation.^23^ Moreover, mitogen-stimulated assembly and nuclear localization of cyclin D1-CDK4 complexes was perturbed by p21^Cip1^ or p27^Kip1^ deficiency.^21^ Conversely, the reintroduction of p21^Cip1^ or p27^Kip1^ reversed the inhibitory effect on the activities of cyclin D-CDK holoenzymes and resulted in increased nuclear levels of active D-cyclin/CDKs to phosphorylate retinoblastoma protein (Rb), thereby promoting cell cycle progression and S-phase entry.^21,24^ However, the involvement of these two cyclin-dependent kinase inhibitors (CDKIs) in LN-521-induced HPC proliferation and their roles in governing the activities of cyclin-CDK complexes have not been investigated.

In the present study, LN-521 was found to be the most efficient culture substrate for the proliferation of HPCs. Moreover, the activation of p53 was found to be essential for LN-521-stimulated HPC proliferation. Activated p53 promoted CDK4 kinase activity and Rb phosphorylation depended on p27^Kip1^ induction. LN-521 induced p53 activation via the Integrin α6β1/FAK-Src-Paxillin/Akt axis. Overall, our findings offer first insights into the positive role of p53 in the proliferation of HPCs promoted by LN-521.

## Results

### Upregulation of p53 is accompanied by increased levels of laminin α4, α5, β2, β1 and γ1 during the proliferative response of HPCs

**Fig. 1:**
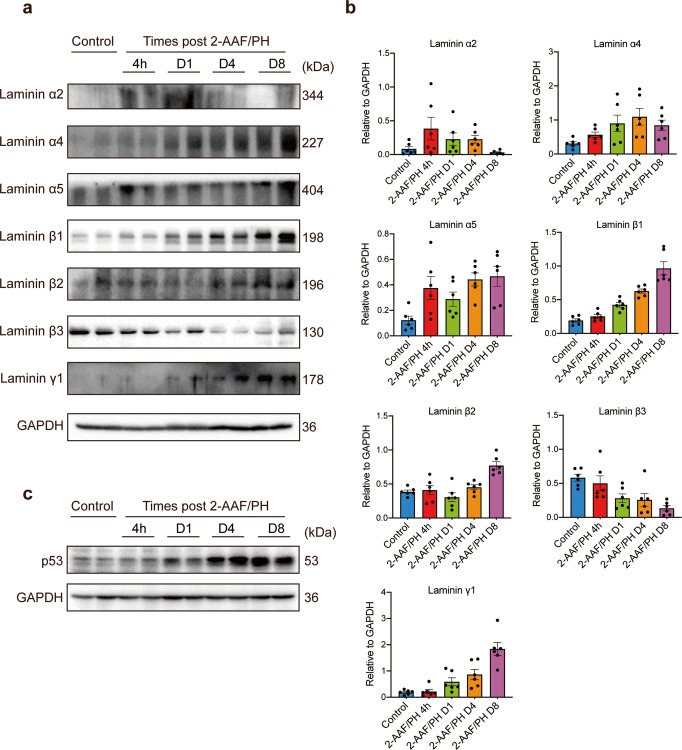
Protein expression of laminin subunits and p53 in the liver of Fischer 344 rats exposed to 2-acetylaminofluorene/partial hepatectomy (2-AAF/PH). **a** Protein expression of laminin α2, α4, α5, β1, β2, β3, and γ1 subunits in the liver of sham-operated rats and 2-AAF/PH rats at different time points post PH was evaluated by western blot analysis. The protein level of GAPDH was used as a loading control. **b** The graphs show the quantification of protein levels normalized to GAPDH and are expressed as means ± SEM (*n* = 6 per group). **c** Protein expression of p53 in the liver of sham-operated rats and 2-AAF/PH rats at different time points post PH (*n* = 6 per group) was evaluated by western blot analysis, and the protein level of GAPDH was used as a loading control

Dense laminins matrix are deposited around HPCs to support their expansion and the maintenance of an undifferentiated phenotype.^4^ However, the exact trimeric composition of the laminins involved in the HPC response has not been elucidated. In this study, the transcriptional changes of laminin constituents were examined and a rapid burst in the transcription levels of *Lama2, Lama4, Lama5, Lamb1, Lamb2, Lamb3* and *Lamc1* was observed at the early stage of HPC response as analyzed by real-time PCR (Supplementary Fig. S1a). We then further detected the protein expression of these laminin subunits (Fig. 1a, b). Laminin α2, which was not detectable in control rats, showed immediate downregulation after 4 h and was barely detectable at day 8 post hepatectomy, while the β3 subunit was highly expressed in control rats and gradually decreased within 8 days. Laminin α4, β1 and γ1 were only weakly expressed in untreated rats, while increasing upregulation was seen from 24 h to 8 days after PH. Laminin α5 exhibited a strong increase of protein expression immediately after PH (within 4 h) and was detected at even higher levels at day 8. Laminin β2 was normally expressed at a very low level in control rats, compared to a significant increase from 4 to 8 days post hepatectomy. The levels of p53 were also measured. As shown in Fig. 1c, in the extracts from control (sham-operated) rats, the p53 level was very low, while 2-AAF/PH treatment induced a pronounced, gradual increase, beginning at day 1 and extending to 8 days. These results suggest that the high levels of α4, α5, β2, β1 and γ1-containing laminins and p53 together are likely to be associated with increased proliferation potential of HPCs.

### LN-521 maximized rat HPC proliferation while maintaining the progenitor phenotype by interacting with α6β1 integrin

**Fig. 2:**
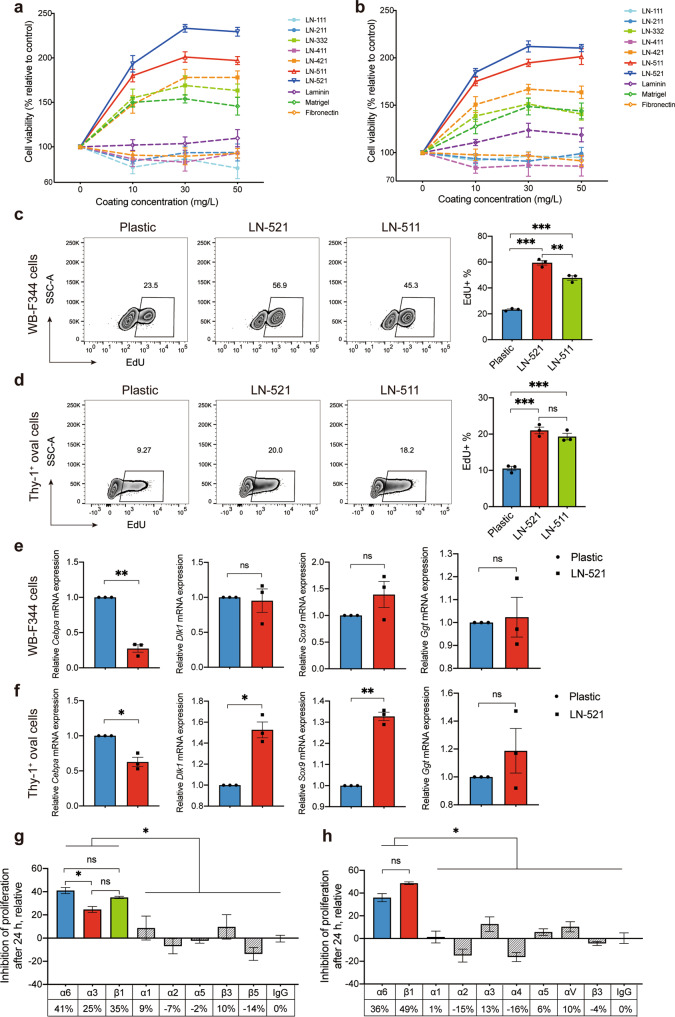
LN-521 maximized the proliferation of rat HPCs while maintaining the progenitor phenotype by interacting with α6β1 integrin. Dose-response proliferation curves of WB-F344 cells (**a**) and Thy-1^+^ oval cells (**b**) on various ECM proteins. The graphs represent the relative number of proliferating cells according to the CCK-8 assay, normalized against the values on an uncoated plastic plate. The data are expressed as means ± SEM of three independent experiments. In **a**, *p* < 0.05 LN-521 vs. other substrates except LN-511 at the concentration of 10 mg/l. ns not significant LN-521 vs. LN-511 at the concentration of 10 mg/l. *p* < 0.05 LN-521 vs. other substrates at the concentration of 30 or 50 mg/l according to one-way ANOVA followed by Tukey’s post hoc test. In **b**, *p* < 0.05 LN-521 vs. other substrates except LN-511 at the concentration of 10, 30 or 50 mg/l. ns not significant LN-521 vs. LN-511 at the concentration of 10, 30 or 50 mg/l according to one-way ANOVA followed by Tukey’s post hoc test. WB-F344 cells (**c**) and Thy-1^+^ oval cells (**d**) were plated on plastic, LN-521 or LN-511, and the proliferation activity was assessed using the EdU incorporation assay. Data are representative of three independent experiments. Flow cytometry analysis of the percentage of EdU^+^ cells was expressed as means ± SEM. ns not significant, **p < 0.01, and ***p < 0.001 according to one-way ANOVA followed by Tukey’s post hoc test. Expression profile of hepatocyte, biliary phenotype and hepatic progenitor markers analyzed by real-time qPCR in WB-F344 cells (**e**) and Thy-1^+^ oval cells (**f**) following culture on plates coated with LN-521 or uncoated plastic plates. The relative mRNA expression of *Cebpa, Dlk1, Sox9* and *Ggt* was normalized to the control gene *Gapdh*, and the data are expressed as fold enrichment values with reference to the levels on an uncoated plastic plate. Experiments were performed in triplicate and data are expressed as means ± SEM. ns not significant, *p < 0.05, **p < 0.01 according to paired Student’s *t* test. Proliferation-blocking experiments, inhibition of the viability of WB-F344 cells (**g**) and Thy-1^+^ oval cells (**h**) on LN-521 by different integrin antibodies. Bars represent inhibition by antibodies against the subunits indicated below the graph. IgG was used as a control. Experiments were performed in triplicate and data are expressed as means ± SEM. ns not significant, *p < 0.05 according to one-way ANOVA followed by Dunnett’s post hoc test

We next determined the proliferative effects of LN-521, LN-511, LN-421, and LN-411 as cell culture matrices, also in comparison with other laminin isoforms (-111, -211, -332, -411 and -511) or other major liver ECM components or mimics (matrigel and fibronectin). As shown in Fig. 2a, b, when WB-F344 cells were seeded onto LN-521, the maximal cell viability increased 2.33-fold compared to uncoated controls, while incubation on LN-521 coated plates increased the maximal viability of Thy-1^+^ oval cells 2.12-fold compared to uncoated controls. The cell viability appeared to be reduced when WB-F344 cells were plated onto LN-111, LN-211, LN-411 or fibronectin, while it remained the same on laminin (defined as a mixture of laminin isoforms). There was an increase of viability in Thy-1^+^ oval cells cultured on laminin, but not in Thy-1^+^ oval cells grown on fibronectin, and there was even a decrease on LN-111, LN-211 and LN-411. LN-511, LN-421, LN-332 and matrigel were capable of supporting WB-F344 and Thy-1^+^ oval cell proliferation, but with less potency than LN-521. The effect of LN-521 on the cell proliferation rate was further assessed by performing flow cytometry following EdU staining. The results indicated that there were more actively DNA-replicating WB-F344 cells (Fig. 2c) and Thy-1^+^ oval cells (Fig. 2d) on plates coated with LN-521 compared to uncoated controls. Moreover, there was a statistically significant increase in the proportion of DNA synthesis in WB-F344 cells grown on LN-521 compared to LN-511. Thy-1^+^ oval cells also exhibited more DNA synthesis on LN-521 than on LN-511, but the difference was not statistically significant.

After culturing on LN-521 or control substrate, cells were harvested and the gene expression of a panel of markers for hepatocyte, biliary and hepatic progenitor phenotype was detected by qRT-PCR. In WB-F344 cells, LN-521 inhibited the expression of the early hepatocyte genes *Cebpa*, while having no significant effect on hepatic progenitor (*Sox9, Dlk1*) and biliary (*Ggt*) markers compared to the control culture (Fig. 2e). A similar expression pattern of *Cebpa* and *Ggt* was observed in Thy-1^+^ oval cells. By contrast, the gene expression of *Sox9* and *Dlk1* was upregulated in Thy-1^+^ oval cells cultured on LN-521(Fig. 2f). These results indicated that LN-521 supported the culture of HPCs in an undifferentiated phenotype.

Integrins were reported to be the main receptors for laminin in the liver,^25^ but the specific integrin involved in the interaction of HPCs with LN-521 was unknown. Firstly, we applied flow cytometry to examine the integrin receptors present on WB-F344 cells and Thy-1^+^ oval cells. As shown in Supplementary Fig. S2a, the α3, α6 and β1 integrins were abundantly expressed on WB-F344 cells, and Thy-1^+^ oval cells expressed high levels of integrin α3, α5, α6 and β1 (Supplementary Fig. S2b). Other integrin subunits only showed weak or none significant expression on both cell lines. Proliferation-blocking experiments with a library of integrin antibodies were further applied and the results showed that the antibody against β1 integrin had an obvious inhibitory effect on the viability of both WB-F344 cells (35%) and Thy-1^+^ oval cells (49%) cultured on LN-521 (Fig. 2g, h). The antibody against α6 integrin also had a significant inhibitory effect on both cell lines (41% for WB-F344 and 36% for Thy-1^+^ oval cells). Blockade of α3 integrin inhibited the effect of LN-521 in WB-F344 cells by about 25%, but did not significantly affect Thy-1^+^ oval cells. Antibodies against other integrin subunits showed very little, if any, effect in either cell line. These results suggested that α6β1 and α3β1 seemed to be the main isoforms involved in the interaction of WB-F344 cells with LN-521, while for Thy-1^+^ oval cells, α6β1 was the main receptor. Overall, LN-521 showed a robust positive effect on HPC proliferation in an undifferentiated phenotype through integrin α6β1 engagement.

### p53 inactivation inhibited HPC proliferation promoted by LN-521

**Fig. 3:**
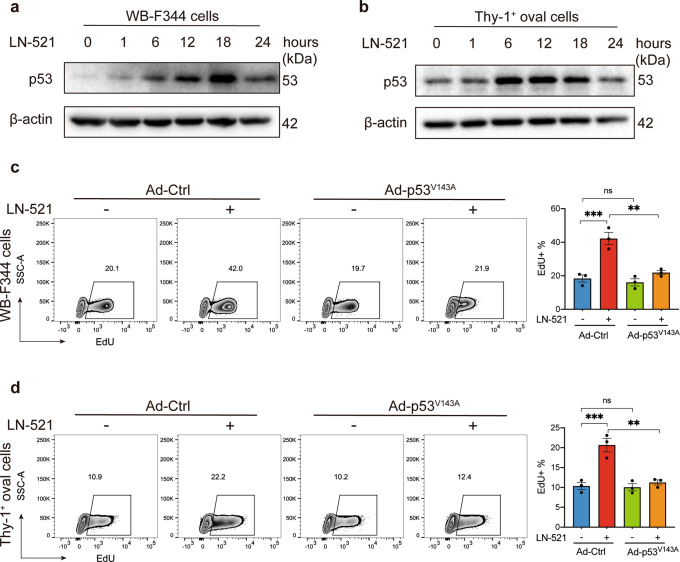
p53 inactivation inhibited HPC proliferation promoted by LN-521. The expression of p53 protein in WB-F344 cells (**a**) and Thy-1^+^ oval cells (**b**) plated on LN-521 for 0, 1, 6, 12, 18 and 24 h was evaluated by western blot analysis. The protein level of β-actin was used as a loading control. The blots shown are representative of three experiments with similar results. WB-F344 cells (**c**) and Thy-1^+^ oval cells (**d**) were infected with Ad-p53^V143A^ or empty adenoviral vector (Ad-Ctrl) 24–36 h prior to seeding on plates coated with LN-521 or uncoated plastic, and the proliferation activity was analyzed using the EdU incorporation assay. Data are representative of three independent experiments. Flow cytometry analysis of the percentage of EdU^+^ cells was expressed as means ± SEM. ns not significant, **p < 0.01, and ***p < 0.001 according to one-way ANOVA followed by Tukey’s post hoc test

In line with its traditional role as a proliferation “brake” that inhibits cellular S-phase entry, p53 should be inhibited under LN-521 stimulation whereby HPC proliferation was significantly induced. However, p53 showed a low abundance in LN-521-free Thy-1^+^ oval cells and WB-F344 cells, and to our surprise, was upregulated when HPCs were grown on LN-521 (Fig. 3a, b). More strikingly, there was a significant decrease in cell proliferation when both cell types were additionally treated with pifithrin-α (PFT-α) (Supplementary Fig. S3), a pharmacologically developed inhibitor of p53 that inhibits its downstream transcriptional targets both in vitro and in vivo. To further confirm the effect of p53 inactivation on the proliferation of HPCs, cells were transfected with an adenoviral vector containing a dominant-negative p53 mutant (Ad-p53^V143A^) which specifically prevents p53 transcriptional activity.^26^ The empty adenoviral vector was included as a negative control (Ad-Ctrl). As shown in Fig. 3c, d, the positive effect of LN-521 on DNA synthesis was abolished when both cell types were forced to express the mutant p53^V143A^. These results suggest the necessity of p53 activation for the promotion of HPC proliferation by LN-521.

### p53 inactivation inhibited LN-521-induced nuclear translocation of CDK4 and inactivation of Rb

**Fig. 4:**
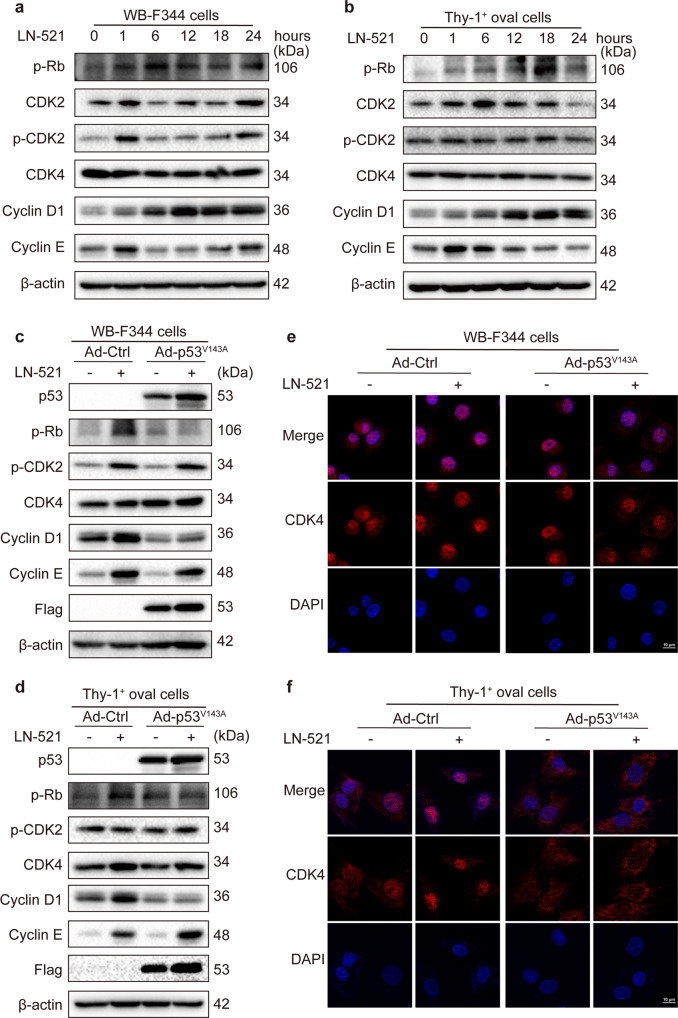
p53 inactivation inhibited the nuclear translocation of CDK4 and Rb inactivation. The protein levels of cyclin D1, cyclin E, p-CDK2, CDK2, CDK4 and p-Rb in WB-F344 cells (**a**) and Thy-1^+^ oval cells (**b**) plated on LN-521 for 0, 1, 6, 12, 18 and 24 h were evaluated by western blot analysis. **c**–**f** WB-F344 cells and Thy-1^+^ oval cells were infected with Ad-p53^V143A^ or empty adenoviral vector (Ad-Ctrl) 24–36 h prior to seeding on plates coated with LN-521 or uncoated plates. **c**, **d** The protein levels of p53, p-Rb, cyclin D1, cyclin E, p-CDK2 and CDK4 in both cell lysates were assessed by western blot analysis. The Flag peptide tag was used as a marker confirming the infection with Ad-p53^V143A^. In **a**–**d**, the protein level of β-actin was used as a loading control, and the blots shown are representatives of three experiments with similar results. Intracellular localization of CDK4 kinase in WB-F344 cells (**e**) and Thy-1^+^ oval cells (**f**) was determined by immunofluorescence staining. The confocal images shown here are representatives of three experiments with similar results (scale bar, 10 μm; magnification, ×63 Zoom 2)

Cyclin-dependent kinases CDK2 and CDK4, which are usually activated in complex with their partners cyclins E and D1, respectively, inactivate retinoblastoma protein (Rb) which leads to the release of E2F transcription factor to initiate DNA replication and S-phase progression.^27^ Accordingly, we first examined the expression of proteins involved in cell cycle control following culture on LN-521. As shown in Fig. 4a, b, the amount of p-Rb protein in WB-F344 cells increased between 1 and 12 h of LN-521 culture followed by a second wave of induction after 18 h. Rb phosphorylation was practically undetectable in Thy-1^+^ oval cells in the absence of LN-521, but increased gradually with prolonged culture. Cyclin E expression was detectable in LN-521-free WB-F344 cells and Thy-1^+^ oval cells, and rapidly increased as early as 1 h following plating on LN-521. In WB-F344 cells, the levels of p-CDK2 showed a tendency similar to cyclin E, with a peak of induction in response to LN-521 after 1 h, whereas in Thy-1^+^ oval cells p-CDK2 levels were independent of LN-521 stimulation and stayed low throughout the 24 h period. In both cell types, the expression of cyclin D1 protein gradually increased under the influence of LN-521, similar to that of p53. CDK4 levels in both WB-F344 cells and Thy-1^+^ oval cells were relatively stable during the 24 h of culture on LN-521.

We then examined the effect of p53 inactivation on these cell cycle factors. After transfection with Ad-p53^V143A^, the phosphorylation of Rb induced by LN-521 was significantly reduced in both cell types (Fig. 4c, d). By contrast, cyclin E and p-CDK2 were still at high levels in HPCs expressing p53^V143A^, suggesting that induction of cyclin E/CDK2 and p53 protein may represent two separate pathways that contribute to LN-521-induced proliferation of HPCs. Conversely, the level of cyclin D1 was downregulated in cells expressing p53^V143A^, whereas the CDK4 protein level was unchanged. We next applied immunofluorescence staining to examine the intracellular localization of CDK4. Ad-Ctrl-infected lines derived from both cell types displayed an intense and exclusive nuclear staining pattern for CDK4 when cultured on LN-521. By contrast, CDK4 was only observed in the cytoplasm of LN-521-stimulated Thy-1^+^ oval cells expressing p53^V143A^, whereas in Ad-p53^V143A^-infected WB-F344 cells CDK4 localized diffusely to the cytoplasm and the nucleus with few cells showing predominantly nuclear staining (Fig. 4e, f). These results suggest that p53 activation is essential for the nuclear translocation of CDK4 kinase and consequent Rb phosphorylation induced by LN-521.

### p53 inactivation inhibited the induction of p27^Kip1^

**Fig. 5:**
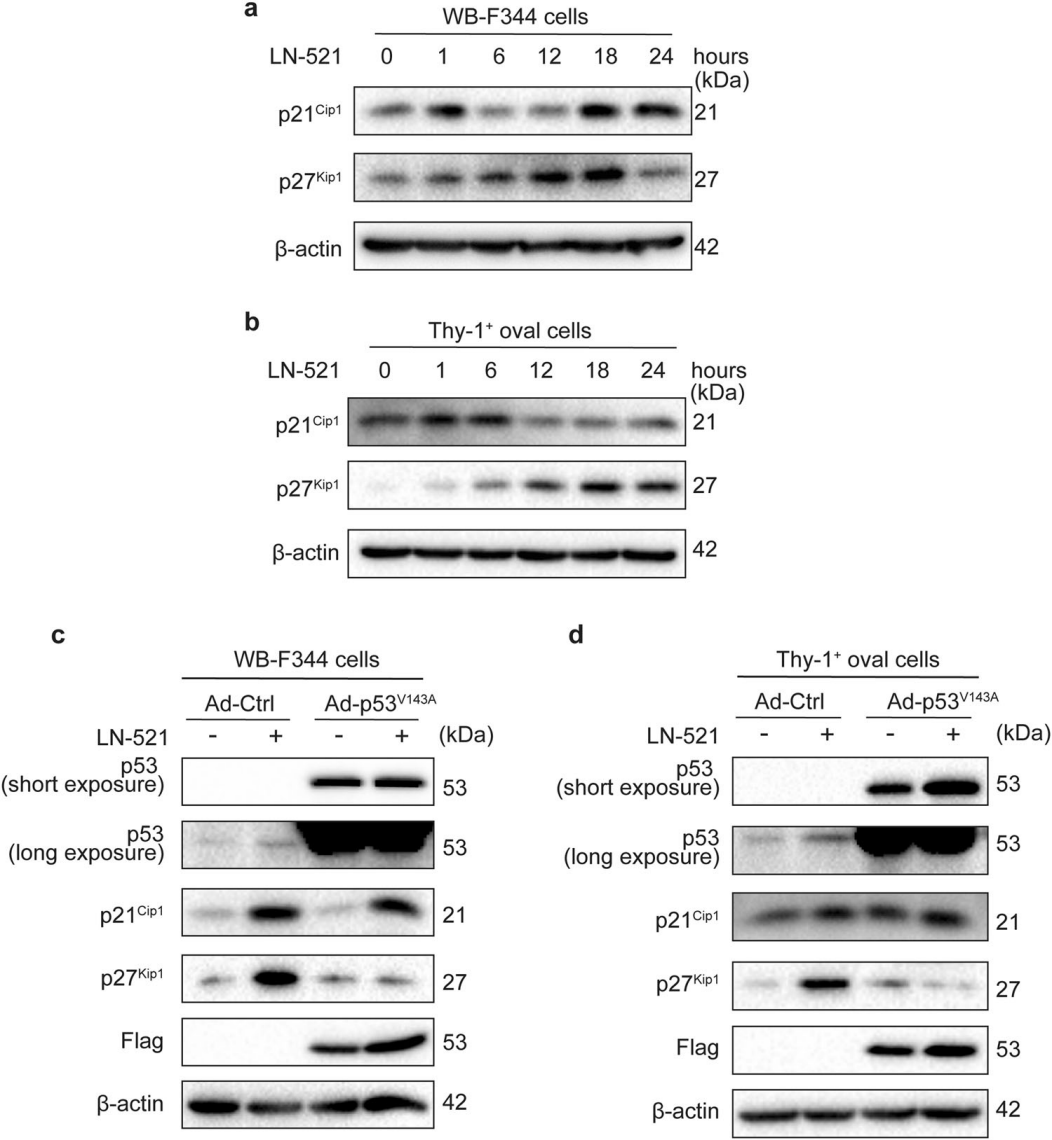
p53 inactivation inhibited the induction of p27^Kip1^. The protein levels of p21^Cip1^ and p27^Kip1^ in WB-F344 cells (**a**) and Thy-1^+^ oval cells (**b**) plated on LN-521 for 0, 1, 6, 12, 18 and 24 h were evaluated by western blot analysis. WB-F344 cells (**c**) and Thy-1^+^ oval cells (**d**) were infected with Ad-p53^V143A^ or empty adenoviral vector (Ad-Ctrl) 24–36 h prior to seeding on plates coated with LN-521 or uncoated plastic. Western blot analysis of p53, p21^Cip1^ and p27^Kip1^ protein was performed on lysates from both cell types. For p53 protein, both the expression of p53 mutants (short exposure) and the endogenous level (long exposure) were detected. The Flag peptide tag was used as a marker confirming the infection with Ad-p53^V143A^. The protein level of β-actin was used as a loading control, and the blots shown are representatives of three experiments with similar results

The p21^Cip1^ and p27^Kip1^ proteins are the two main CDKIs that act downstream of p53 in regulating cell cycle progression. We studied the expression patterns of these two proteins. As shown in Fig. 5a, b, p27^Kip1^ expression in both WB-F344 cells and Thy-1^+^ oval cells were LN-521 dependent, and increased in a trend that was analogous to that of p53. p21^Cip1^ was weakly expressed in both cell types in the absence of LN-521 stimulation, and in WB-F344 cells the expression of p21^Cip1^ was significantly increased within 1 h of culture on LN-521, whereas in Thy-1^+^ oval cells it was upregulated within 6 h of treatment with LN-521. We next sought to determine whether the inactivation of p53 may interfere with the induction of p21^Cip1^ or p27^Kip1^. As shown in Fig. 5c, d, the amounts of p21^Cip1^ did not vary substantially in p53 mutant cells than that of p53 wild-type WB-F344 cells and Thy-1^+^ oval cells following LN-521 treatment. By contrast, p27^Kip1^ was markedly suppressed in LN-521-stimulated WB-F344 cells expressing p53^V143A^, and p27^Kip1^ induction in Thy-1^+^ oval cells were almost completely abrogated when cells were exposed to Ad-p53^V143A^ in the presence of LN-521. These results suggest that p27^Kip1^, rather than p21^Cip1^, may act downstream of p53 in promoting HPC proliferation induced by LN-521.

### Ectopic p27^Kip1^ expression restored the nuclear accumulation of CDK4 and S-phase entry

**Fig. 6:**
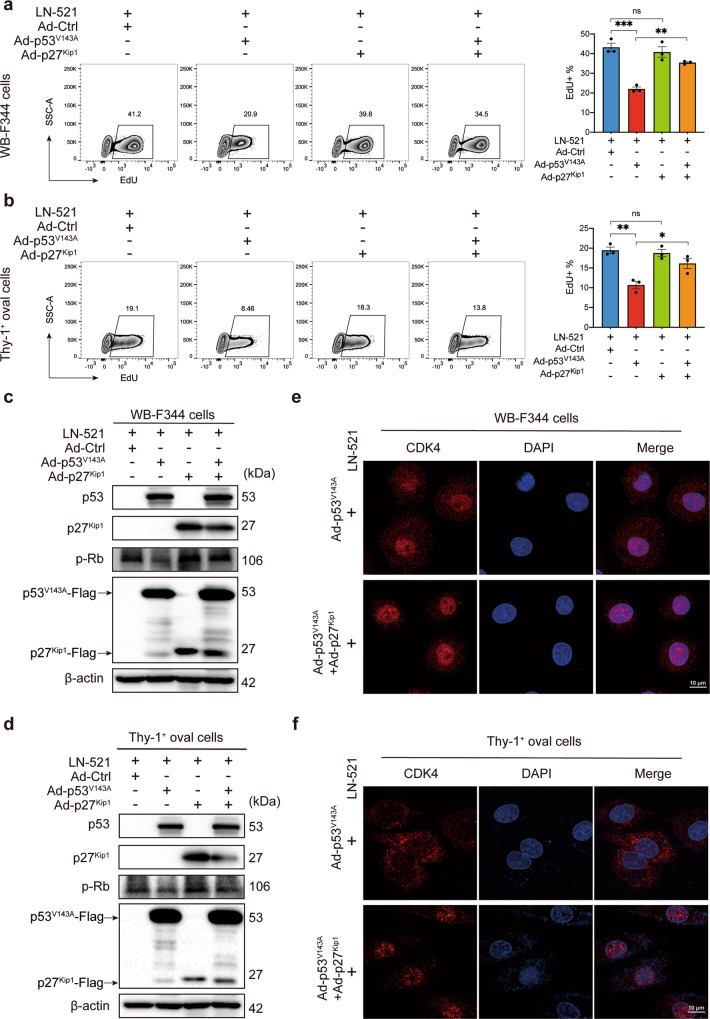
Ectopic p27^Kip1^ expression restored the nuclear accumulation of CDK4 and S-phase entry. WB-F344 cells and Thy-1^+^ oval cells were infected with an empty adenoviral vector (Ad-Ctrl), the mutant Ad-p53^V143A^, the wide-type Ad-p27^Kip1^, or co-infected with Ad-p53^V143A^ and Ad-p27^Kip1^ 24–36 h prior to seeding on plates coated with LN-521. **a**, **b** The proliferation activity was analyzed using the EdU incorporation assay. Data are representatives of three independent experiments. Flow cytometry analysis of the percentage of EdU^+^ cells was expressed as means ± SEM. ns not significant, *p < 0.05, **p < 0.01, and ***p < 0.001 according to one-way ANOVA followed by Tukey’s post hoc test. **c**, **d** Western blot analysis of p53, p27^Kip1^ and p-Rb protein levels was performed on lysates from both cell types. The Flag peptide tag was used as a marker confirming infection with Ad-p53^V143A^ or Ad-p27^Kip1^, and the protein level of β-actin was used as a loading control. The blots shown are representatives of three experiments with similar results. The intracellular location of CDK4 kinase in WB-F344 cells (**e**) and Thy-1^+^ oval cells (**f**) was determined by immunofluorescence staining. The confocal images shown are representatives of three independent experiments with similar results (scale bar, 10 μm; magnification, ×63 Zoom 2)

To further determine whether the inhibitory effects of p53^V143A^ on HPC proliferation could be rescued by reintroduction of p27^Kip1^, an adenoviral vector encoding the wild-type p27^Kip1^ protein (Ad-p27^Kip1^) was used. Co-expression of p27^Kip1^ in both cell types significantly reversed the inhibitory effect of the mutant p53^V143A^ on LN-521-induced DNA synthesis (Fig. 6a, b). Furthermore, the decrease of Rb phosphorylation in Ad-p53^V143A^ HPCs was restored following reintroduction of p27^Kip1^ (Fig. 6c, d). In contrast to the cells expressing p53^V143A^, dense nuclear accumulation of CDK4 reappeared when p53^V143A^ Thy-1^+^ oval cells were co-infected with Ad-p27^Kip1^ and similar results were seen in WB-F344 cells (Fig. 6e, f). Therefore, the results suggest that p27^Kip1^, which acts downstream of p53, is involved in the activation of CDK4 kinase and the inactivation (phosphorylation) of Rb.

### p53 was activated by LN-521 via the Integrin α6β1/FAK-Src-Paxillin/Akt axis

**Fig. 7:**
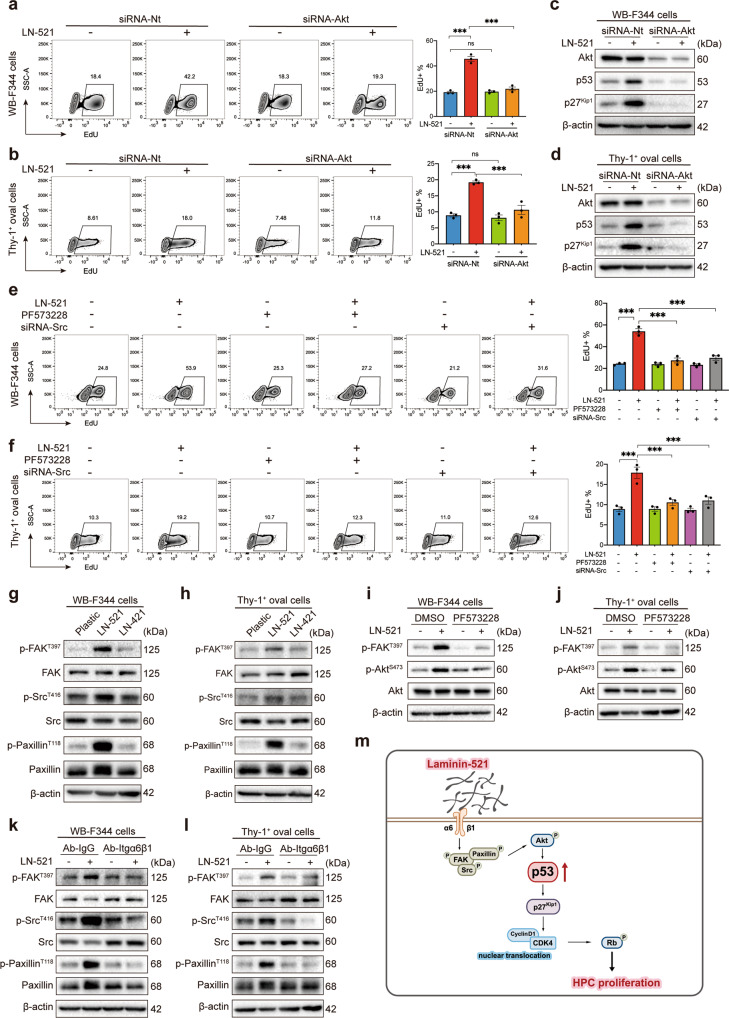
p53 was activated by LN-521 via the Integrin α6β1/FAK-Src-Paxillin/Akt axis. **a**, **b** WB-F344 cells and Thy-1^+^ oval cells were transfected with siRNA against Akt or a control scramble siRNA for 36–48 h prior to seeding on plates coated with LN-521 or uncoated plastic. The proliferation activity of WB-F344 cells (**a**) and Thy-1^+^ oval cells (**b**) was analyzed using the EdU incorporation assay. Data are representatives of three independent experiments. Flow cytometry analysis of the percentage of EdU^+^ cells was expressed as means ± SEM. ns not significant, ***p < 0.001 according to one-way ANOVA followed by Tukey’s post hoc test. Western blot analysis of p53 and p27^Kip1^ levels was performed on lysates from WB-F344 cells (**c**) and Thy-1^+^ oval cells (**d**) transfected with siRNA targeting Akt or control duplex for 36–48 h prior to seeding on plates coated with LN-521 or uncoated plastic. The protein level of β-actin was used as a loading control, and the blots shown are representatives of three experiments with similar results. **e**, **f** WB-F344 cells and Thy-1^+^ oval cells were pretreated with PF-5732228 (5 μM) for 1 h or transfected with siRNA against Src for 36–48 h prior to seeding on plates coated with LN-521 or uncoated plastic. The proliferation activity of WB-F344 cells (**e**) and Thy-1^+^ oval cells (**f**) was analyzed using the EdU incorporation assay. Data are representatives of three independent experiments. Flow cytometry analysis of the percentage of EdU^+^ cells was expressed as means ± SEM. ***p < 0.001 according to one-way ANOVA followed by Tukey’s post hoc test. Representative immunoblots for FAK/p-FAK^Tyr397^, Src/p-Src^Tyr416^ and Paxillin/p-Paxillin^Tyr118^ in WB-F344 cells (**g**) and Thy-1^+^ oval cells (**h**) grown on plates coated with LN-521, plates coated with LN-421, or uncoated plastic plates. Representative immunoblots for p-FAK^Tyr397^ and Akt/p-Akt^Ser473^ performed on lysates from WB-F344 cells (**i**) and Thy-1^+^ oval cells (**j**) with DMSO or PF573228 (5 μM) pretreatment for 1 h prior to seeding on plates coated with LN-521. Representative immunoblots for FAK/p-FAK^Tyr397^, Src/p-Src^Tyr416^ and Paxillin/p-Paxillin^Tyr118^ performed on lysates from WB-F344 cells (**k**) and Thy-1^+^ oval cells (**l**) pretreatment with an inactivating antibody against α6β1 integrin for 30 min prior to seeding on plates coated with LN-521 or uncoated plastic plates. **g**–**l** The protein level of β-actin was used as a loading control, and the blots shown are representatives of three experiments with similar results. **m** Schematic of the signaling pathways through which LN-521 promoted the proliferation of HPCs. LN-521 binds α6β1 integrin to sequentially activate FAK-Src-Paxillin cascade and Akt signaling, which subsequently leads to the upregulation of p53, which enhances the activation of CDK4 and inactivation of Rb by inducing p27^Kip1^, which leads to the proliferation of HPCs

It was reported that p53 levels are closely regulated by cell proliferation signals via the PI3K/Akt and MEK/ERK pathways.^11,14^ Our data showed that blockade of PI3K/Akt activity using the specific inhibitor LY294002 abrogated the proliferation of WB-F344 cells and Thy-1^+^ oval cells. By contrast, treatment with PD98059, a specific inhibitor of MEK/ERK, did not have effect on the proliferation of the two cell types in the presence of LN-521 (Supplementary Fig. S4). Our data also showed that the phosphorylation of Akt at Ser473 induced by LN-521 treatment was inhibited by LY294002 in both WB-F344 cells and Thy-1^+^ oval cells, suggesting its blocking of PI3K/Akt activity (Supplementary Fig. S5). The cells were further transfected with Akt-siRNA to specifically interfere with Akt signaling (Supplementary Fig. S6), and DNA synthesis was also reduced (Fig. 7a, b). Moreover, the induction of p53 and p27^Kip1^ by LN-521 was abrogated in both cell types when Akt pathway was abolished (Fig. 7c, d). Thus, we excluded the possibility of the involvement of the ERK pathway and confirmed that activation of Akt was necessary for the LN-521 dependent sequential induction of p53 and p27^Kip1^ in proliferating HPCs.

Akt can be activated by multiple signals, including focal adhesion kinase (FAK)-Src, integrin-linked kinase (ILK) or ras‐associated protein-1 (Rap1), some of which may transduce integrin activation to Akt by recruiting PI3K.^28–31^ To determine which signal may be involved in the activation of Akt during the proliferation by LN-521, HPCs were treated with specific inhibitors or siRNAs targeting FAK-Src, ILK, Rap1 or left untreated. Among these intervention strategies, there were significant reductions in DNA synthesis promoted by LN-521 when both cell types were treated with the FAK inhibitor PF573228 or the siRNA directed against Src (Fig. 7e, f and Supplementary Fig. S7). By contrast, inhibition of ILK or Rap1 did not have any inhibitory effect on cell proliferation (Supplementary Fig. S8). Moreover, FAK activation was evidenced by a greater increase of phosphorylation at Tyr397 when both cell lines were grown on LN-521 than on LN-421 or uncoated plastic (Fig. 7g, h). A higher phosphorylation of Src^Tyr416^ and Paxillin^Tyr118^ was also observed in both cell types on LN-521 than on LN-421 or uncoated plastic, in agreement with the view that the FAK-Src complex can target the adapter protein Paxillin.^28^ Moreover, the induction of Akt phosphorylation (Fig. 7i, j) by LN-521 was inhibited by the FAK inhibitor PF573228. We further confirmed that blockade of integrin signal transduction using deactivating antibodies against integrin α6β1 inhibited activation of FAK-Src-Paxillin complex in LN-521 cultured WB-F344 cells and Thy-1^+^ oval cells (Fig. 7k, l). In summary, these data suggest that PI3K/Akt is activated by a signal from integrin α6β1 bound to LN-521, which is transduced via the FAK-Src-Paxillin cascade. In this context, p53 appears to be activated by LN-521 through the Integrin α6β1/FAK-Src-Paxillin/Akt axis to induce its downstream effector p27^Kip1^, which subsequently drives the nuclear translocation of CDK4 kinase and Rb phosphorylation to induce the proliferation of HPCs (Fig. 7m).

### p53 inactivation reduced the proliferation of HPC transplants in vivo

**Fig. 8:**
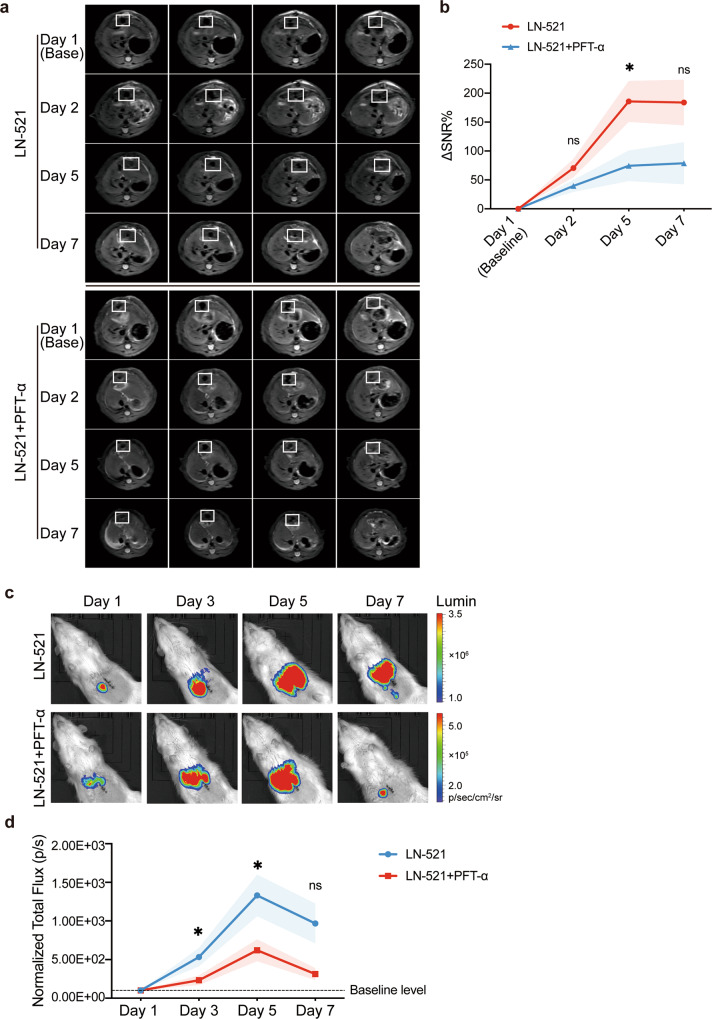
p53 inhibition reduced the proliferation of HPC transplants in vivo. **a**, **b** WB-F344-USPIO cells were transplanted into rat livers on the day of PH and serial in vivo MRI in rats receiving cells delivered in LN-521 substrates, with or without added PFT-α, was performed on the day after transplantation (day 1, also the Baseline) and following 2, 5 and 7 days (*n* = 6 per group). **a** Shown are representative sets of T2-weighted MR images in rats from each group; white frames indicate the hypointense regions consistent with the injected cells. **b** ΔSNR% based on the T2 sequence are shown as means ± SEM. ns not significant and *p < 0.05 vs. LN-521 + PFT-α group according to Mann–Whitney *U* test. **c**, **d** WB-F344-luc cells were transplanted into rat livers on the day of PH and bioluminescence imaging of the rats receiving cells delivered in LN-521 substrates, with or without added PFT-α, was performed on the day of transplantation (day 1, also the Baseline) and following 3, 5, and 7 days. **c** Representative bioluminescence images are shown and **d** Normalized Total flux (p/s) are shown as means ± SEM. There were 14 rats per group on day 1, followed by a loss and there were 12 rats in the LN-521 group or 12 rats in the LN-521 + PFT-α group left on day 7. ns not significant, *p < 0.05 vs. LN-521 + PFT-α group according to Mann–Whitney *U* test

To ascertain the role of p53 in the positive effect of LN-521 on the proliferation capacity of HPCs in vivo, WB-F344 cells delivered in LN-521 substrates, with or without added PFT-α, were transplanted into the liver of experimental rats. To track these cells in vivo, WB-F344 cells were pre-labeled with USPIO (named WB-F344-USPIO), and over 90% of cells successfully incorporated USPIO particles as confirmed by Rhodamine B, Prussian blue iron staining and transmission electron microscopy (TEM) (Supplementary Fig. S9). In both T2- and T2*-weighted MRI scans, the hypointense signals from WB-F344-USPIO cells were detectable on the day of transplantation (day 1, also the Baseline) and persisted in both group of rats for 7 days (Fig. 8a and Supplementary Fig. S10). MRI signal intensity analysis based on the T2 sequence demonstrated that the signals gradually increased in the first few days in both groups, but to a different extent. At 5 days after transplantation, the ΔSNR% values showed a statistically significant reduction in rats treated with LN-521 + PFT-α compared to LN-521 group rats (Fig. 8b).

Luciferase-transfected WB-F344 cells (named WB-F344-luc) were utilized to further confirm the involvement of p53 in the promotion of HPC proliferation by LN-521 in vivo. During the consecutive observation, rats from both groups had a detectable BLI signal at every time point (Fig. 8c). As shown in Fig. 8d, there was a higher total flux at each time point in both groups relative to the baseline level, which indicated the proliferation of cells in both groups. A statistically significant reduction of total flux was seen in the LN-521 + PFT-α group compared to the LN-521 group from day 3 to day 5, suggesting a lower rate of proliferation in WB-F344-luc cells following p53 inhibition. Taken together, these results further confirm that p53 is necessary for the pronounced proliferation of HPCs promoted by LN-521.

## Discussion

This is, to our knowledge, the first demonstration that p53 is upregulated when HPCs are driven to proliferate. Ohlson et al. analyzed the expression pattern of p53 following 2AAF-induced hepatocyte growth inhibition, and found an upregulation of hepatic p53 protein levels.^32^ In combination with our findings showing an increase of hepatic p53 expression during the process of HPC proliferation induced by 2AAF/PH, we suspected that p53 may simultaneously be involved in both hepatocyte growth inhibition and HPC proliferation as two separate processes. These data are in line with earlier studies showing that HPCs are permitted to extensively proliferate only when hepatocytes are completely inhibited or extremely ablated.^1^ Notably, the upregulation of p53 protein was accompanied by the elevation of α5, β2 and γ1 laminin chains, which constituted the LN-521 isoform that provided a more robust substratum for HPC proliferation not enabled by other laminin isoforms in the present study. Moreover, we found that the LN-521-induced increase in the levels of p53 protein, which did not exhibit a variation in its transcriptional levels (Supplementary Fig. S11), was dependent on Akt pathway in HPCs. It was reported that Akt could inhibit the activity of MDM2, either directly or indirectly, to reduce MDM2-mediated p53 ubiquitination, which ultimately leads to an increase of p53 protein stabilty.^33,34^ In agreement with these findings, our results implied the possibility of posttranslational regulation of p53 protein by Akt in HPCs stimulated by LN-521, but this hypothesis requires further investigation.

Strikingly, our present work showed that inactivation of p53 by PFT-α or Ad- p53^V143A^ inhibited HPC proliferation. In support of our finding, a clearly reduced proliferation rate was reported for neural stem cells when p53 was knocked down.^35^ Interestingly, the proliferation of HPCs was also inhibited in this study when wild-type p53 was overexpressed (Supplementary Fig. S12), which strongly suggested that a subtle balance of the p53 dosage is crucial for its role in regulating the proliferation of HPCs. The role of p53 in HPC proliferation was also tested in vivo by innovative combination of MRI and BLI, which allows complementary analysis of cell location and cell proliferation in animals. The MRI measurements showed more hypointense signals decreasing in the LN-521 group than in the LN-521 + PFT-α group, which may indicate that the cell proliferation was partially inhibited by PFT-α. However, the results from MRI analysis alone cannot exclude the interference of cell death as the release of iron particles from dead cells may also contribute to the decrease of the hypointense signals.^36^ Thus, BLI analysis, whose signals provide specific information on the number of viable cells, was also applied. According to the BLI results, the increase of signal intensity observed in both groups was actually resulted from cell expansion. Therefore, the lower extent of change in the LN-521 + PFT-α group was confirmed mainly due to the reduction of cell expansion capacity following p53 inhibition. What’s more, although previous studies showed that the process of HPC proliferation was accompanied by matrix remodeling,^37^ in the present study, we found that the administration of LN-521 did not result in any adverse effects such as liver fibrosis while inducing HPC proliferation (Supplementary Fig. S13). Unfortunately, except for a decrease of serum TBIL levels, it did not show any significant decrease in the levels of serum ALT, AST, ALP, ALB, and γGT (Supplementary Fig. S14), suggesting that liver damage was not significantly alleviated by transplantation of oval cells in combination with LN-521. We speculate that this is due to the limited supplementation of LN-521, the cell transplantation approach, and the short observation period. Although the follow-up was limited to 7 days and the cell fate after a longer period needs further investigation, we confirmed that p53 inhibition prevented the proliferation of transplanted HPCs. Conversely, loss of p53 led to an increased proliferative response of HPCs in CDE treated p53^−/−^ mice.^38^ Thus, the inconsistency of these results strongly supported the idea that p53 may have a dual role in regulating stem cell proliferation according to differences in timing, cell types, stimulus, and even types of model, which determined the status of p53 and its targeted effectors.

The cyclin E-CDK2 complex is one of the potential targeted effectors of p53 in regulating DNA synthesis. The expression of Cyclin E as well as the activation of CDK2 was examined and showed unchanged when p53 was inactivated whereby the suppression of Rb activity was abolished. The effects observed here suggested that Rb phosphorylation induced by p53 was independent of Cyclin E-CDK2. This is in agreement with previous findings that cyclin E can activate DNA synthesis even in the absence of Rb phosphorylation.^39,40^ Thus, we hypothesized that although it may be involved in LN-521-induced DNA synthesis in HPCs, the induction of cyclin E-CDK2 by LN-521 was independent of p53 signaling. Cyclin D1-CDK4 is another important targeted effector of p53 in regulating cell-cycle progression. It has been proven that cyclin D1-CDK4 kinase alone is sufficient to initiate DNA synthesis, inducing quiescent cells to enter the S-phase.^41^ For a long time, the mechanism was not well understood until very recently Topacio et al. found that cyclin D1-CDK4 could specifically target Rb for phosphorylation by recognizing it via C-terminal helix-based docking.^24^ Phosphorylated Rb then dissociates from E2F and thereby drives the expression of E2F-target genes that initiate DNA replication, driving cell-cycle progression from the G1 to the S-phase.^42^ In this context, the increased nuclear translocation of CDK4 kinase and subsequent DNA synthesis are prevented by p53 inactivation. Based on these findings, p53 potentially activates cyclin D1-CDK4, which sequentially phosphorylates Rb protein that commits cells to pass the restriction point just prior to S-phase entry in proliferating HPCs stimulated by LN-521.

In this study, we found a simultaneous LN-521-dependent induction of p53 and p21^Cip1^. Because its promoter contains p53-responsive elements that can be transcriptionally regulated by p53, p21^Cip1^ is now widely accepted to be a key factor for CDK activation downstream of p53.^43^ Beyond our anticipation, the LN-521 induced upregulation of p21^Cip1^ was unchanged whether p53 was inactivated or not, making it unlikely that p21^Cip1^ contributes to p53-mediated CDK4 activation. Notably, our findings showed that a sequential induction of p27^Kip1^ followed by p53 plays an essential role in the regulation of CDK4 activity. This raises the possibility that p27^Kip1^ induction in HPCs requires p53 function, and similar results were obtained with the introduction of DNA binding domain mutants such as p53^C135Y^, p53^R175H^ or p53^R248W^, which abolished the increase of *p27* transactivation,^44^ although the molecular mechanisms underlying p53-dependent regulation of *p27* are diverse. Canonically, the transcriptional activity of p53 relies on binding to its response elements located in the promoter region of the target genes.^45^ Moreover, binding of endogenous p53 to the *p27* promoter in the region from -417 to -199 has also been suggested,^46^ although it lacks a canonical DNA binding sequence for p53 protein. This has been recently explained by the possibility of p53 interacting with the putative decameric half-site residing in the *p27* promoter in a non-canonical way.^44^ Overall, our results suggest that activation of the p53-p27^Kip1^ pathway, but not the p53-p21^Cip1^ pathway, is essential for LN-521-induced S-phase entry. This puts forward a novel mechanism for the regulation of cell-cycle progression by p53.

Based on these findings, we hypothesized that p27^Kip1^ also played a positive role in the regulation of cell-cycle progression by inducing the nuclear translocation of CDK4. It is well-known that cyclin D1 binds to CDK4, forming a kinase complex that translocates into the nucleus before activation.^47^ However, the cyclin D1-CDK4 complex does not appear to associate readily. It has been suggested that p27^Kip1^ may function as an assembly factor for this unstable complex that enhances cyclin D1-CDK4 kinase activity by improving the stability of the protein complex.^20^ Furthermore, D-type cyclins and CDK4 lack nuclear localization signals (NLSs), which are needed for the nuclear entry of the complex. Thus, the putative NLSs located at the C-terminus of p27^Kip1^ may mediate the nuclear import of the cyclin D1-CDK4 complex after binding.^21^ Moreover, as an intrinsically disordered protein, p27^Kip1^ shows high conformational flexibility, which raises the possibility of it exerting special functions, including the activation of CDK.^48,49^ This was further elucidated in a recent study reporting that p27^Kip1^ allosterically activates CDK4 in complex with cyclin D1 to promote the phosphorylation of Rb.^50^ However, further studies are needed to clarify the mechanisms through which p53 regulates p27^Kip1^ and the latter in turn regulates cyclin D1-CDK4 in HPCs stimulated by LN-521.

Taken together, this study identified LN-521 as an optimal substrate with great stimulatory effects for the propagation of HPCs and furthermore revealed a previously-unexpected positive role of p53 in the regulation of HPC proliferation, based on its induction of p27^Kip1^-dependent CDK4-activation and promotion of DNA synthesis. These findings provide novel insights into p53 as a potential target for the genetic manipulation of stem cells to enhance their proliferation and maintenance ability for effective tissue regeneration.

## Materials and methods

### Animals

Male Fischer 344 rats weighing 140–160 g were obtained from the Institute of Experimental Animal Research, Chinese Academy of Medical Sciences (Beijing, China). They were housed in a specific pathogen-free facility under a 12-h light/dark cycle with free access to food and water. All animal experiments were conducted in accordance with the “Guide for the Care and Use of Laboratory Animals” (NIH publication 86-23, revised 1985), and were approved by the Committee on the Ethics of Animal Experiments of Tongji Hospital of HUST.

### Cell culture, infection and transfection

Hepatic progenitor cell line WB-F344 was obtained and cultured as described previously.^51,52^ Thy-1^+^ oval cells were isolated and cultured as described below. For experiments requiring ECM treatment, 96- or 6-well cell culture plates were pre-coated with sterile solutions of laminins or other matrices at a concentration of 10–50 mg/l overnight at 4 °C prior to cell seeding. DPBS containing Mg^2+^ and Ca^2+^ was used to dilute laminins and fibronectin. DMEM/IMDM was used to dilute matrigel. Human recombinant laminin isoforms were purchased from BioLamina, laminin was purchased from Sigma, matrigel and fibronectin were obtained from Corning. After washing three times with PBS, the pre-coated plates were blocked with BSA for 1 h at 37 °C, washed twice with PBS, and washed once with serum-free culture medium just before cells were added. The remaining laminin solution could be reused once, and the cell behavior experiments were carried out using fresh coating materials.

For overexpression analyses, both WB-F344 cells and Thy-1^+^ oval cells were infected with flag-tagged Ad-p53(V143A) adenovirus (DesignGene Biotechnology, Shanghai, China), which coded for a dominant-negative p53 mutant of Val → Ala substitution at position 143, the flag-tagged Ad-p27^Kip1^ adenovirus (Vigene Biosciences, Jinan, China), which corresponded to the full-length *p27* gene, and the empty adenoviral vectors (Ad-Ctrl) as the control. Cells were cultured for at least 36 h post transfection before further experiments were performed. Prior studies have shown that adenoviruses infect HPCs with high efficiency, and under the conditions studied here, over 90% of HPCs were transfected with Ad-p53^V143A^ or Ad-p27^Kip1^.

For siRNA-mediated gene silencing, both cell types were transfected with siRNAs for 36–48 h using Lipofectamine 3000 transfection reagent (Thermo Fisher Scientific) according to the manufacturer’s instructions. Transfection of non-silencing siRNA was used as a negative control. To rule out potential off-target effects of siRNAs, three pairs of siRNAs for each gene were designed as follows:

Control: 5’-TTCTCCGAACGTGTCACGTdTdT-3’,

Src-1: 5’-GGCTTACTACTCCAAACAT-3’,

Src-2: 5’-CTGACCTGTCCTTCAAGAA-3’,

Src-3: 5’-CAGAAGAACCCATTTACAT-3’,

Akt-1: 5’-GGCCCAACACCTTCATCAT-3’,

Akt-2: 5’-CCCACACGCTTACTGAGAA-3’,

Akt-3: 5’-GCTGTTCGAGCTCATCCTA-3’.

Specific silencing efficacy was confirmed by western blot analysis.

### Inhibitors

Where indicated, chemicals such as FAK inhibitor PF573228 (MedChem Express), PI3K/Akt inhibitor LY294002 (MedChem Express), Erk inhibitor PD98059 (MedChem Express), p53 inhibitor PFT-α (Selleck Chemicals), ILK inhibitor QLT0267 (MedChem Express) and Rap1 inhibitor ESI-09 (MedChem Express) were added as indicated to the assays described above.

### Isolation of primary hepatic progenitor cells

To prepare primary hepatic progenitor cells, the 2-AAF/PH procedure was performed to induce HPC activation and expansion as previously described.^53^ Then, the livers were perfused and digested with a two-step protocol at day 10 following PH to yield the greatest number of HPCs. Once isolated, percoll density gradient separation was applied to obtain a pure population of non-parenchymal cells (NPCs), followed by flow cytometry for further isolation of hepatic progenitor cells as described previously.^54,55^ Briefly, purified NPC fractions were sorted into Thy-1^+^ and Thy-1^−^ fractions using a BD FACS flow cytometer after incubation with an FITC-conjugated mouse anti-rat Thy-1 antibody for 45 min. This technique resulted in Thy-1^+^ oval cell populations with a purity of 95 to 97% expressing the hepatic stem cell markers α-fetoprotein, γ-glutamyl transpeptidase, cytokeratin-19, and OV6.^56^ Cell viability was determined by trypan blue exclusion. Thy-1^+^ oval cells were collected in Iscove’s Modified Dulbecco’s Medium (IMDM) supplemented with 10% fetal bovine serum and 0.1% insulin.

### CCK-8 assay

To assess cell viability, serum-starved cells were re-suspended in 100 μl of fresh culture medium containing 0.2% FBS for WB-F344 cells and 10% FBS for Thy-1^+^ oval cells, and cultured in the pre-coated 96-well plates at 37 °C for 24 h. Viable counts were assessed at the indicated time points using the CCK-8 assay according to the manufacturer’s instructions. After a further 60 min or 2 h of incubation with CCK-8 solution, the plate was scanned using an enzyme-linked immunosorbent assay plate reader (Bio-Tek Elx800) at 450 nm.

### EdU incorporation assay

5-ethynyl-20-deoxyuridine (EdU) labeling assay was conducted using the Cell-Light™ EdU Apollo® 488 In Vitro Flow Cytometry Kit (RiboBio, C10338-3). Briefly, after the indicated treatments and incubation with EdU (at the concentration of 1:1000) for 3 h in the case of WB-F344 cells or 8 h in the case of Thy-1^+^ oval cells, the cells were harvested and processed for the flow cytometry analysis on an LSRFortessa flow cytometer (Becton Dickinson) according to the manufacturer’s instructions.

### HPC orthotopic liver transplantation model

A 2-AAF administration in combination with about 50% partial hepatectomy (2-AAF/50% PH) model was established to induce HPC activation^57,58^ (Supplementary Fig. S15) and at the same time, to provide adequate space for cell transplantation. Labeled WB-F344 cells were injected into the remaining livers after hepatectomy as previously described,^59^ and the in vivo fate of implanted oval cell grafts was recorded by both in vivo bioluminescence imaging (BLI) and magnetic resonance imaging (MRI). Rats were euthanized after the continuous observation, and blood and liver tissues were collected. Serum was isolated to detect aspartate aminotransferase (AST), alkaline phosphatase (ALT), albumin (ALB), alkaline phosphatase (ALP), gamma-glutamyl transpeptidase (γ-GT) and total bilirubin (TBIL) using an auto analyzer (Rayto). Liver sections were immunofluorescent stained to determine the retention and hepatic differentiation of transplanted cells within the graft site (Supplementary Fig. S16).

### Magnetic resonance imaging (MRI)

For MRI studies, WB-F344 cells were labeled with Molday ION Rhodamine B (BioPAL, Worcester, MA, USA), an ultrasmall superparamagnetic iron oxide (USPIO) material that is detectable by MR imaging according to the manufacturer’s instructions. The USPIO labeling efficiency was confirmed by TEM, Prussian blue staining, and Rhodamine B immunofluorescence. Then, the USPIO-labeled WB-F344 cells were harvested and suspended in 100 μl of LN-521-containing substrate, with or without added PFT-α. The cells were injected into the remaining liver and viewed by MRI scanning at 1, 2, 5 and 7 days after transplantation. To ensure the transplantation efficiency was similar among animals, cells were injected into the remaining livers at the same position. In vivo MRI was conducted on a 3.0T MR scanner (GE Medical Systems) using a customized animal coil with a diameter of 5 cm wrapped around the abdomen of the rats. T2- and T2*-weighted imaging was performed for each rat with the following parameters. For the T2 sequence, Repetition time: 3000 ms; echo time: 48 ms; slice thickness: 1.5 mm; inter-slice gap: 0 mm; field of view: 80 mm × 80 mm; number of averages: 6; matrix: 192 × 160. For T2* sequence, Repetition time: 400 ms; echo time: 10 ms; slice thickness: 1.5 mm; inter-slice gap: 0 mm; field of view: 80 mm × 80 mm; number of averages: 3; matrix: 256 × 192. Two radiologists with 5 years of experience in abdominal MRI analysis evaluated the T2-weighted images. The regions of interest were manually drawn along the edges of the area with hypointensity on all slices, and the background noise was determined by drawing a region outside the anatomy of the rats. Signal-to-noise ratio (SNR) was calculated by dividing the mean signal intensity of target sites by the standard deviation of the background noise as follows: SNR = SI_target_/SI_noise_. The data were then calculated using the following equation: ΔSNR% = [(SNR_post_ − SNR_base_)/SNR_base_]*100, where SNR_base_ and SNR_post_ correspond to the SNR of baseline (day 1) and days post transplantation, respectively. This was done to allow for a direct comparison of how the hypointense signals changed over time, as well as to remove the variable of differences in initial cell distribution.

### Bioluminescence imaging (BLI)

For in vivo BLI, the rats were inoculated with luciferase-bearing WB-F344 cells suspended in 100 μl of LN-521-containing substrate, with or without PFT-α added. Luciferase bioluminescence of each rat was detected at 1, 3, 5 and 7 days after transplantation, respectively. On the day of imaging, each rat was intraperitoneally injected with 150 mg/kg of the substrate D-luciferin (Gold Biotechnology, USA) and anesthetized by 1% sodium pentobarbital. Imaging was performed within 15 min after luciferin injection using a Xenogen IVIS three-dimensional small-animal in vivo imaging system and analyzed using Living Image software. The region of interest was selected around each abdomen and the total flux (photos/sec) was calculated. Then the BLI signals was normalized for each rat individually with the total flux of the first time point imaging (day 1, also the Baseline) being 100%. We chose to normalized the data to remove the variable of differences in initial cell distribution and to allow for a direct comparison of how the signal changes over time.

### Immunofluorescent staining

To analyze the nuclear translocation of CDK4, cells grown on pre-coated dishes with indicated treatments were fixed in 4% paraformaldehyde, permeabilized with 0.2% Triton X-100 in PBS, blocked with 2% BSA, and incubated with a primary antibody overnight at 4 °C. Then, the cells were incubated with fluorescently labeled secondary antibodies at room temperature for 1.5 h, followed by counterstaining of the nuclei with DAPI for 5 min in the dark. The images were acquired using a Zeiss LSM 710 confocal microscope and Zen software with an oil-immersion Plan-Apochromat ×63 NA 1.4 objective (Carl Zeiss Microscopy GmbH), and lasers 405 nm (DAPI) and 561 nm (CDK4). For fluorescently labeled immunohistochemistry, paraffin sections (3 μm-thick) were rehydrated, permeabilized with 0.1% Triton X-100, blocked with 5% goat serum and stained with the primary antibodies overnight at 4 °C. Subsequently, fluorescently labeled secondary antibodies were used to visualize the primary antibodies. Images were scanned digitally using Pannoramic MIDI (3DHISTECH) and analyzed using CaseViewer software.

### Histochemistry and immunohistochemistry

Fresh isolated liver tissues were fixed with 4% paraformaldehyde, embedded in paraffin, and cut into sections of 3 μm thickness. After deparaffinization with xylene and rehydration, H&E staining was performed according to standard techniques to determine HPC response. Sirius Red staining was applied to evaluate collagen deposition. For immunohistochemistry analysis, the polymer HRP detection system (Zhongshan Goldenbridge Biotechnology) was used. Briefly, liver paraffin sections were deparaffinized, rehydrated, subjected to antigen retrieval, and blocked with 5% BSA for 1 h, followed by immunostaining with the primary antibody against α-SMA overnight at 4 °C. After washing, the sections were incubated with the HRP conjugated secondary antibody for 45 min at room temperature. Detection was performed using DAB, followed by counterstaining with hematoxylin. The slides were scanned digitally using NanoZoomer (Hamamastu) and analyzed using NDP. View software.

### Analysis of integrin receptors by flow cytometry

To determine the expression of integrins on the surface of WB-F344 cells and Thy-1^+^ oval cells, samples comprising 1 × 10^5^ cells were incubated with the primary antibody, washed in PBS and incubated with the corresponding FITC-conjugated secondary antibody. Fluorescence was detected on an LSRFortessa flow cytometer (Becton Dickinson) and data analysis was performed using FlowJo software (FlowJo, LLC). The antibodies used in this study are listed in Supplementary Table 2.

### Proliferation-blocking assay using integrin antibody

To evaluate the effect of integrin subunits on cell proliferation, cells were incubated with function blocking antibodies against integrin (concentration as recommended by supplier) for 30 min at 37 °C followed by plated onto LN-521-coated plates and subjected to the cell proliferation assay as described above. The antibodies to α2 (559987, BD Biosciences), α3 (sc-7019, Santa Cruz Biotechnology), α4 (553348, BD Biosciences), α6 (sc-59920, Santa Cruz Biotechnology), αv (ab124968, Abcam), and β5 (AB1926, Merck Millipore) integrin subunits were dialyzed using the Zeba™ Spin Desalting Columns (89889, Thermo Fisher Scientific) before use.

### Quantitative real-time PCR analysis

Total RNA was isolated using TRIzol® RNA Isolation Reagents (Thermo Fisher Scientific) according to the manufacturer’s instructions. The reverse transcription was conducted with 1 µg of total RNA using the ReverTraAce cDNA Synthesis kit (TOYOBO). Quantitative real-time polymerase chain reaction (PCR) was performed using a CFX96 Thermal Cycler (Bio-Rad) in 10 µl of reaction mixtures containing cDNA, SYBR Green Real-time PCR Mix (TOYOBO) and primers at final concentrations of 500 nM. For normalization of target gene expression levels, ratios relative to the housekeeping gene GAPDH were calculated using the comparative CT method (ΔΔCT). The primers used for qRT-PCR are listed in Supplementary Table 1.

### Western blot analysis

Cells or tissues were rinsed with ice-cold PBS and lysed in RIPA buffer supplemented with Protease and Phosphatase Inhibitor Mini Tablets (Thermo Fisher Scientific). The protein concentration was determined using a BCA Protein Assay Kit (Thermo Fisher Scientific). Total protein extracts from cell or tissue lysates were denatured, resolved by SDS-polyacrylamide gel electrophoresis and transferred to PVDF membranes (Merck Millipore). After blocking with 5% non-fat milk or BSA, the PVDF membranes were incubated with the primary antibodies overnight at 4 °C, followed by incubation with appropriate secondary antibodies conjugated with horseradish peroxidase. Signals were detected using ECL reagent and digitized using a CCD camera (ChemiDoc, Bio-Rad). The relative intensities of protein bands were analyzed using ImageJ software (National Institutes of Health, Bethesda, MD, USA). Detailed information on the antibodies is listed in Supplementary Table 2.

### Statistical analysis

For in vivo MRI and BLI studies, Mann–Whitney *U* test was used for testing statistical difference between the two groups. For in vitro studies, unless indicated, Student’s *t* test was performed to compare the difference between two groups and one-way ANOVA followed by Tukey’s test was applied to compare multi groups. All analysis was performed using GraphPad Prism version 8.0 (GraphPad Software, Inc., San Diego, CA, USA). *p* < 0.05 was defined as statistically significant.

## Supplementary information

SUPPLEMENTAL MATERIAL

## Data Availability

The data that support the findings of this study are available from the authors upon reasonable request.
