# Supplementary material for: p53 positively regulates the proliferation of hepatic progenitor cells promoted by laminin-521
Source: Signal Transduct Target Ther. 2022 Aug 31;7:290. doi: 10.1038/s41392-022-01107-7 (PMC9427945; doi:10.1038/s41392-022-01107-7)
Supplement: Supplementary file 1 — SUPPLEMENTAL MATERIAL [file 41392_2022_1107_MOESM1_ESM.docx]

Supplementary Materials for

**p53 positively regulates the proliferation of hepatic progenitor cells promoted by laminin-521**

**Author information**

Mingyang Ma^1,2,#^, Shuyao Hua^3,#^, Xiangde Min^4^, Liang Wang^4^, Jun Li^5^, Ping Wu^6^, Huifang Liang^2,7^, Bixiang Zhang^2,7,8^, Xiaoping Chen^2,7,8,*^, Shuai Xiang^2,7,*^

# authors contributed equally to this work.

*Correspondence to: chenxpchenxp@163.com, xiangshuai_hust@163.com

**This PDF file includes:**

Figures. S1 to S16

Tables S1 to S3

Figure. S1.

**
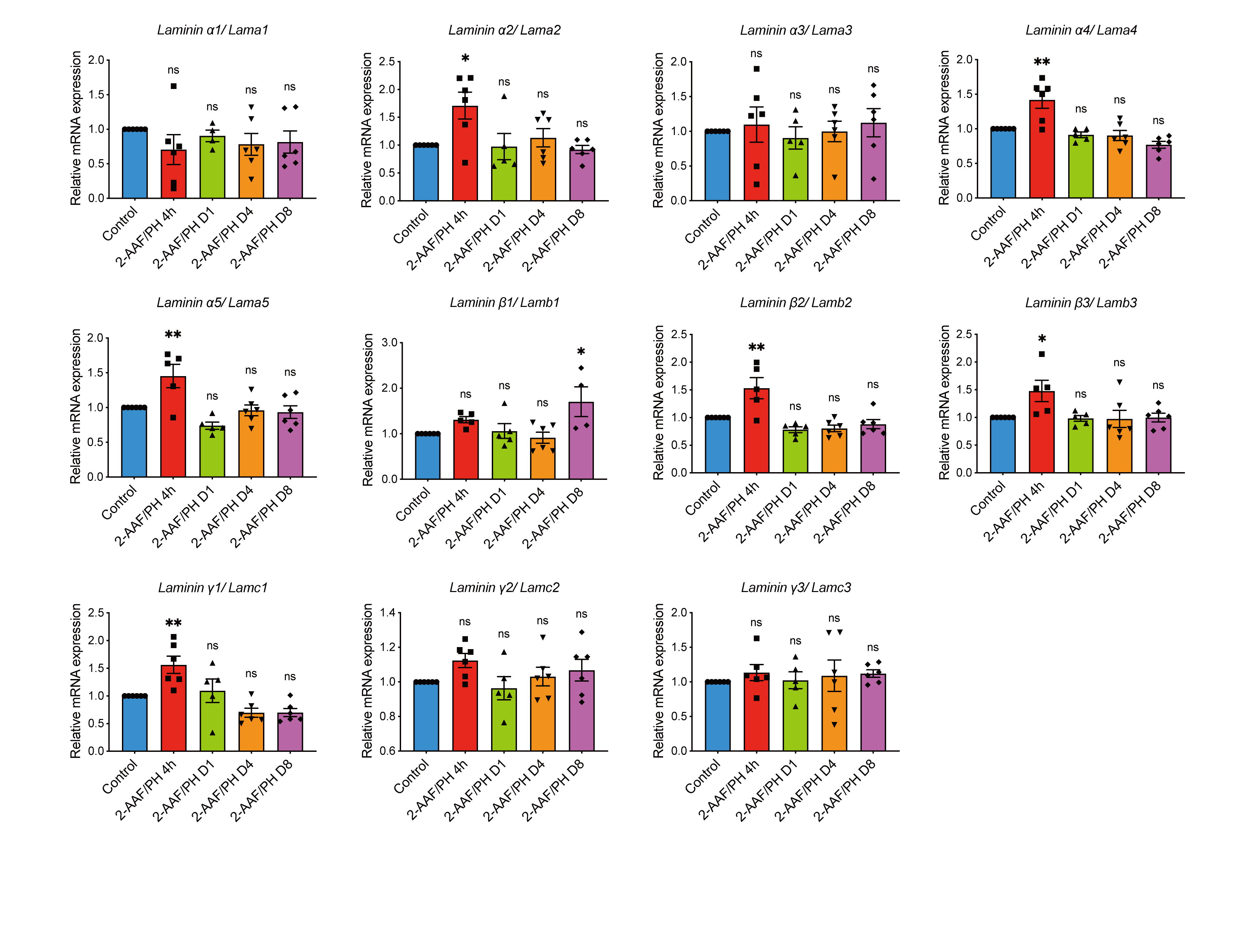
**

**Fig. S1** **Transcriptional levels of different laminin subunits in the liver of Fischer 344 rats following 2-acetylaminofluorene/partial hepatectomy (2-AAF/PH).** Transcriptional levels of laminin subunit genes in the livers of sham-operated rats and rats subjected to 2AAF/PH at different time points (n=5-6 per group) were quantified by real-time PCR. The graphs represent the quantification of mRNA levels of *Lama1, Lama2, Lama3, Lama4, Lama5, Lamb1, Lamb2, Lamb3, Lamc1, Lamc2 and Lamc3* at each time-point normalized to the control gene *Gapdh* with reference to the levels in the sham control (means ± SEM). ns, not significant, **P* < 0.05, and ***P* < 0.01 vs Control according to one-way ANOVA followed by Dunnett's post hoc test.


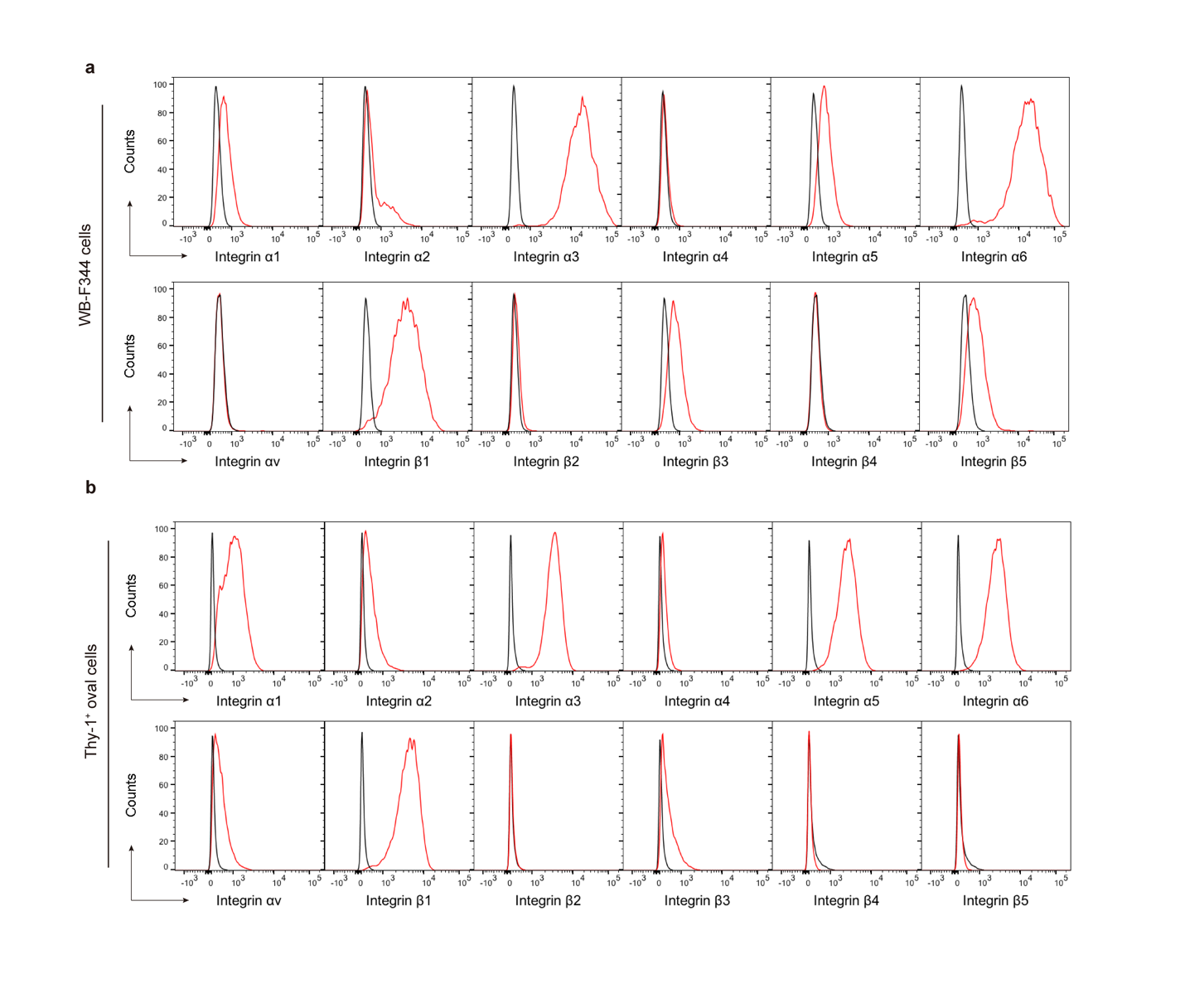
Figure. S2.

**Fig. S2 Phenotypic analysis of hepatic progenitor cell surface integrin receptor expression by flow cytometry.** **a-b** WB-F344 cells (**a**) and Thy-1^+^ oval cells (**b**) were treated with antibodies against α1, α2, α3, α4, α5, α6, αv, β1, β2, β3, β4 and β5 integrin subunits (red profiles) or with the corresponding isotypes (black profiles), followed by incubation with corresponding FITC-conjugated secondary antibody to detect the expression of integrin subunits. Data are representative of two independent experiments.


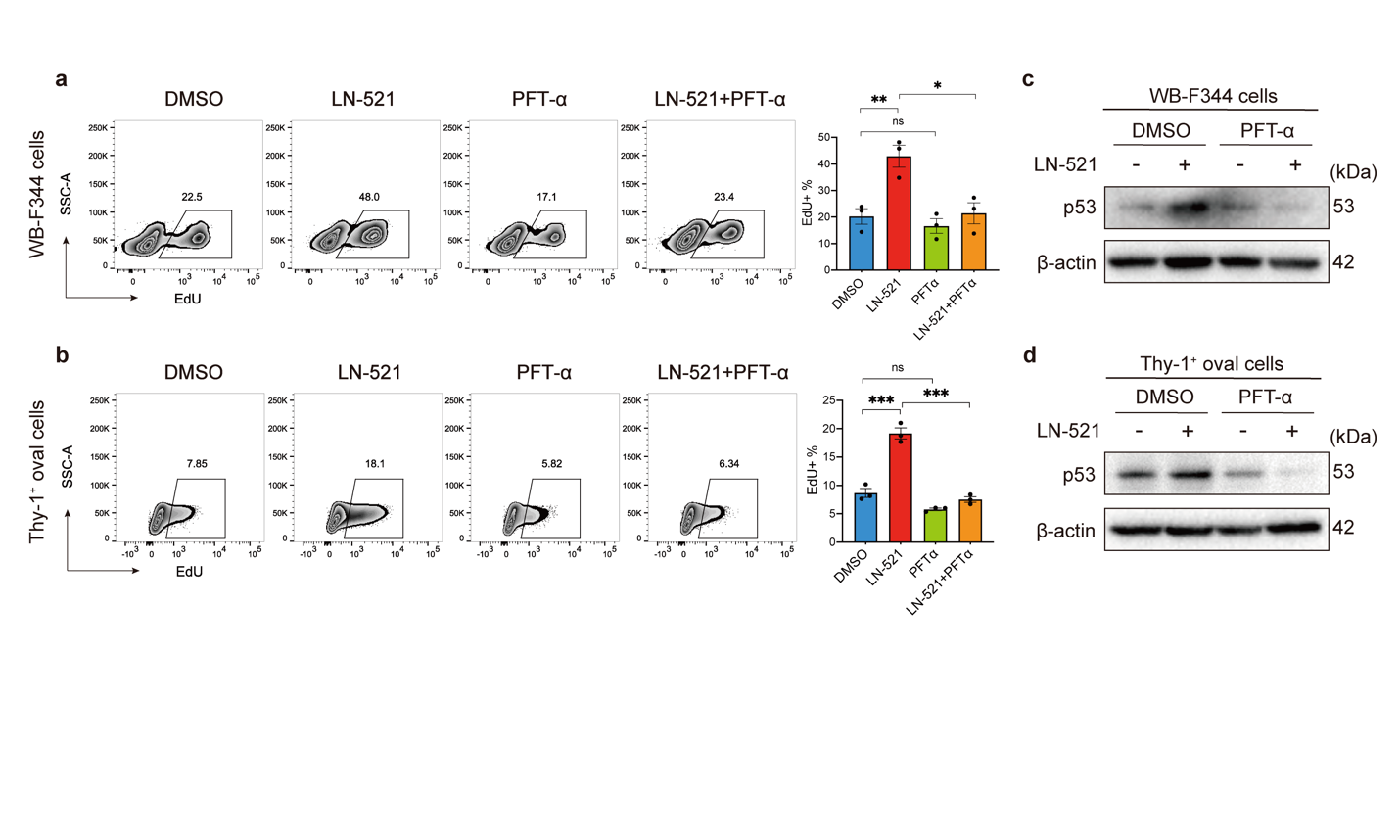
Figure. S3.

**Fig. S3 Reduced proliferation activity in HPCs treated with PFT-α.** WB-F344 cells and Thy-1^+^ oval cells were pretreated with DMSO or PFT-α (5μM) 1h prior to seeding on LN-521 or uncoated plastic. **a-b** The proliferation activity was analyzed using the EdU incorporation assay. Data are representative of three independent experiments. Flow cytometry analysis of the percentage of EdU^+^ cells was expressed as means ± SEM. **P* < 0.05, ***P* < 0.01, and ****P* < 0.001, ns, not significant according to one-way ANOVA followed by Tukey’s post hoc test. **c-d** The protein expression of p53 in WB-F344 cells and Thy-1^+^ oval cells subjected to the indicated treatment was examined by western-blot analysis. The protein level of β-actin was used as a loading control. The blots shown are representatives of three experiments with similar results.


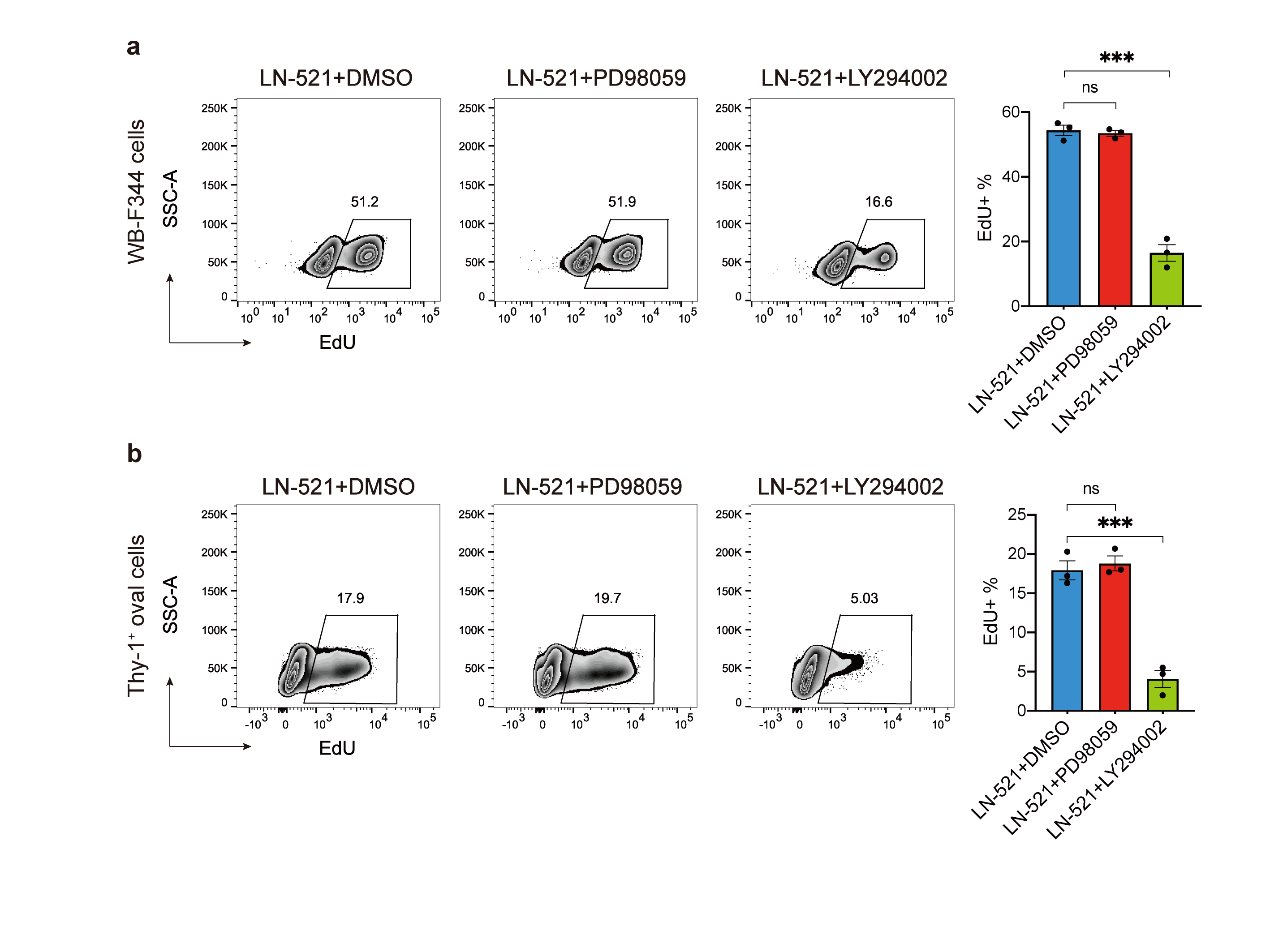
Figure. S4.

**Fig. S4 Effect of Akt or Erk inactivation on the proliferation of HPCs plated on LN-521. a** WB-F344 cells and **b** Thy-1^+^ oval cells were pretreated with DMSO, LY294002 (5μM) or PD98059 (5μM) 1h prior to seeding on LN-521. The proliferation activity was analyzed using the EdU incorporation assays. Data are representative of three independent experiments. Flow cytometry analysis of the percentage of EdU^+^ cell was expressed as means ± SEM. ****P* < 0.001, ns, not significant according to one-way ANOVA followed by Tukey’s post hoc test.


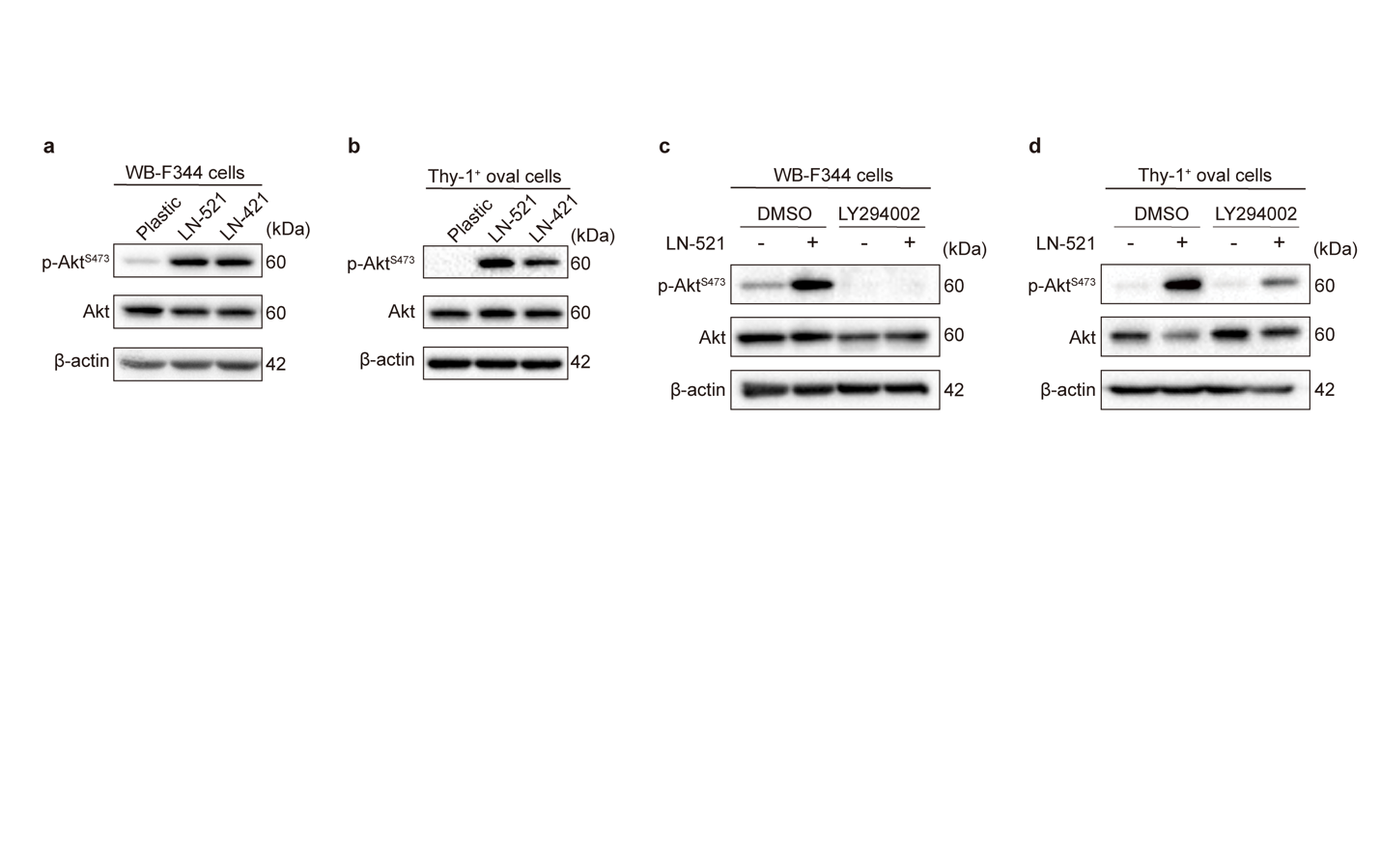
Figure. S5.

**Fig. S5** **Activation of Akt induced by LN-521 was inhibited by LY294002.** **a-b** Western-blot analysis of Akt/p-Akt^Ser473^ in WB-F344 cells (**a**) and Thy-1^+^ oval cells (**b)** grown on plates coated with LN-521, plates coated with LN-421, or uncoated plastic plates. **c-d** Western-blot analysis of total Akt and p-Akt^S473^ protein was performed on lysates from WB-F344 cells (**c**) and Thy-1^+^ oval cells (**d**) pretreated with DMSO or LY294002 (5μM) for 1h prior to seeding on LN-521 or uncoated plastic. The protein level of β-actin was used as a loading control. The blots shown are representatives of three experiments with similar results.

Figure. S6.

**
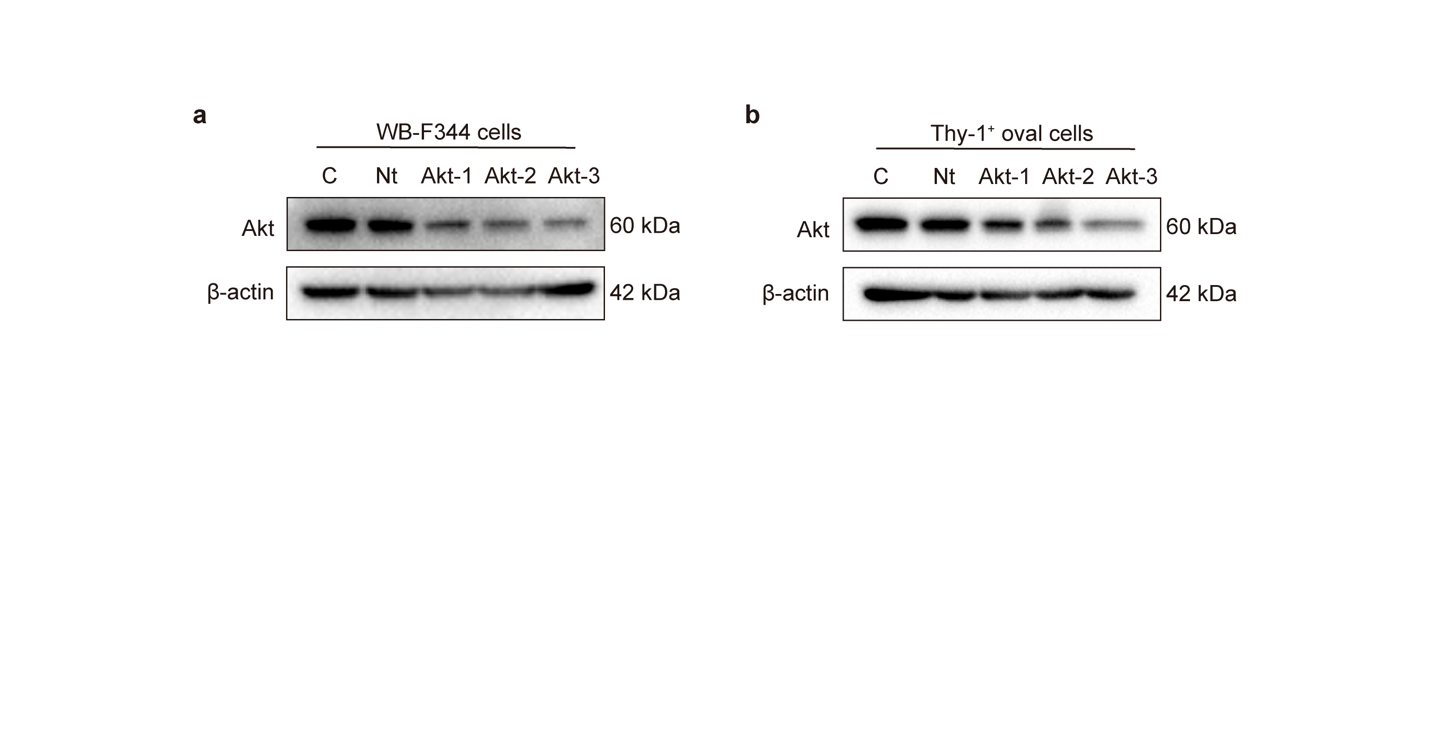
**

**Fig. S6** Western-bolt analysis to confirm the efficacy of siRNAs targeting Akt was performed on lysates from **a** WB-F344 cells and **b** Thy-1^+^ oval cells transfected with three different sequences of Akt siRNA or a scramble siRNA for 36-48h. The protein level of β-actin was used as a loading control.

Figure. S7.

**
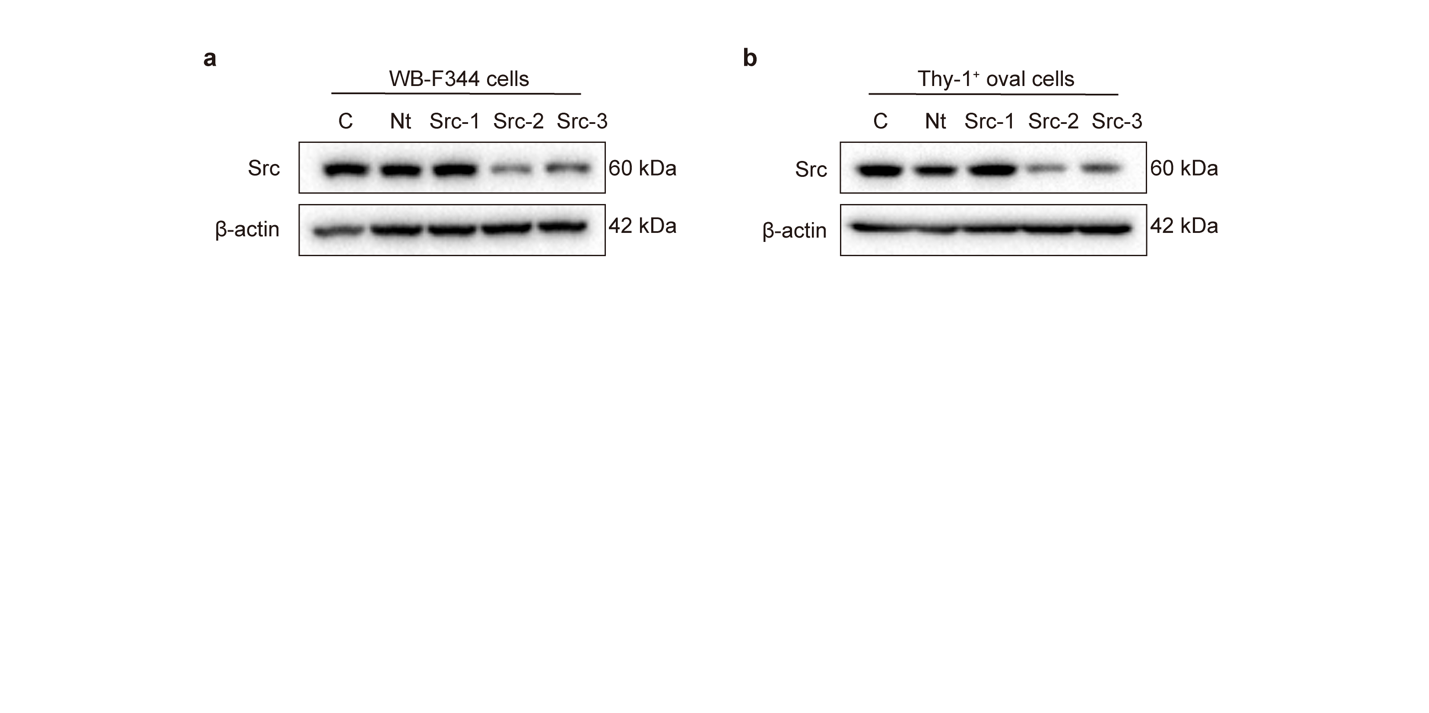
**

**Fig. S7** Western-bolt analysis to confirm the efficacy of siRNAs targeting Src was performed on lysates from **a** WB-F344 cells and **b** Thy-1^+^ oval cells transfected with three different sequences of Src siRNA or a scramble siRNA for 48h. The protein level of β-actin was used as a loading control.

Figure. S8.

**
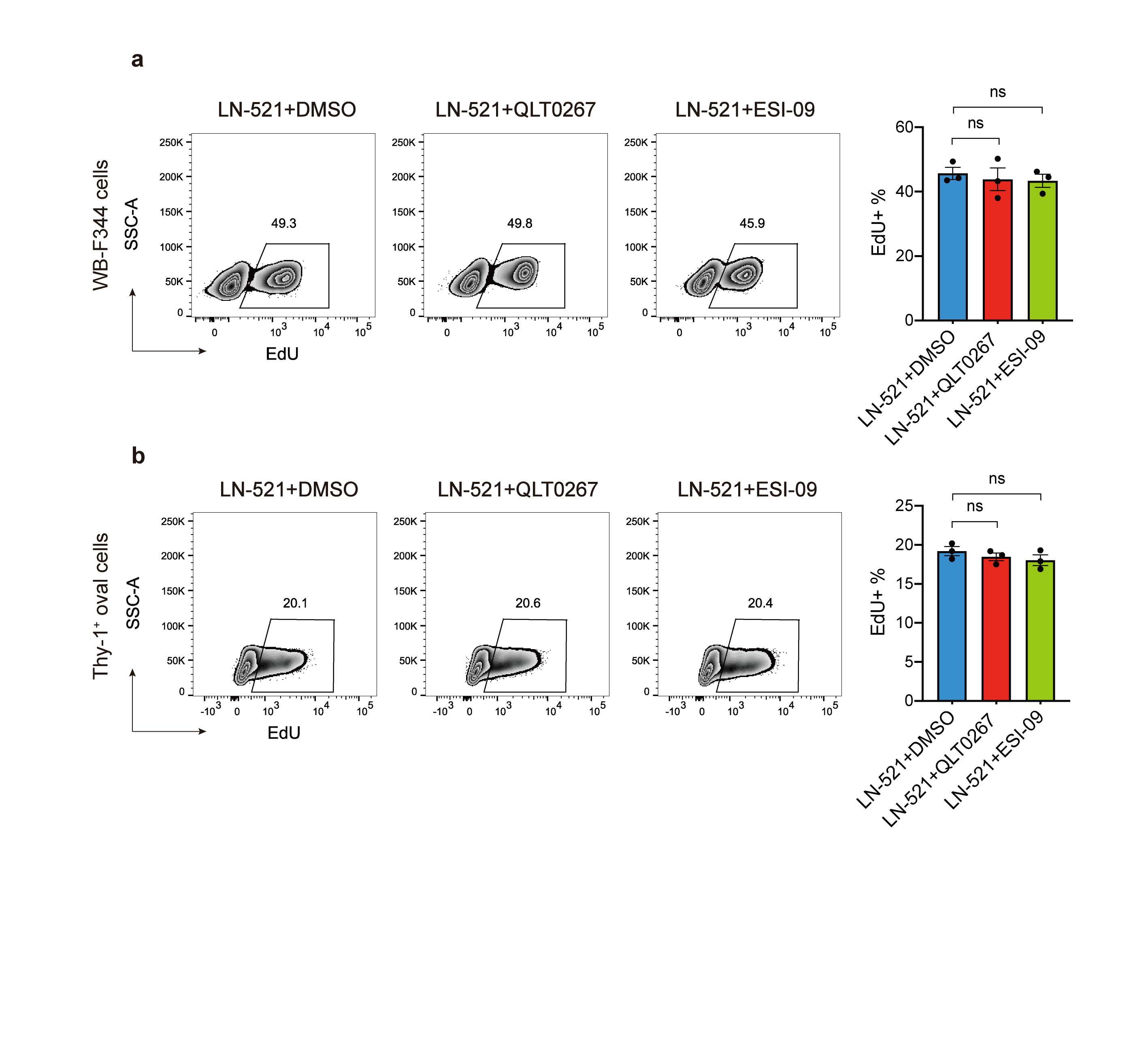
**

**Fig. S8 Effect of ILK or Rap1 inactivation on the proliferation of HPCs plated on LN-521. a** WB-F344 cells and **b** Thy-1^+^ oval cells were pretreated with DMSO, QLT0267 (5μM) or ESI-09 (1μM) 1h prior to seeding on LN-521. The proliferation activity was analyzed using the EdU incorporation assays. Data are representatives of three independent experiments. Flow cytometry analysis of the percentage of EdU^+^ cells was expressed as means ± SEM. ns, not significant according to one-way ANOVA followed by Tukey’s post hoc test.


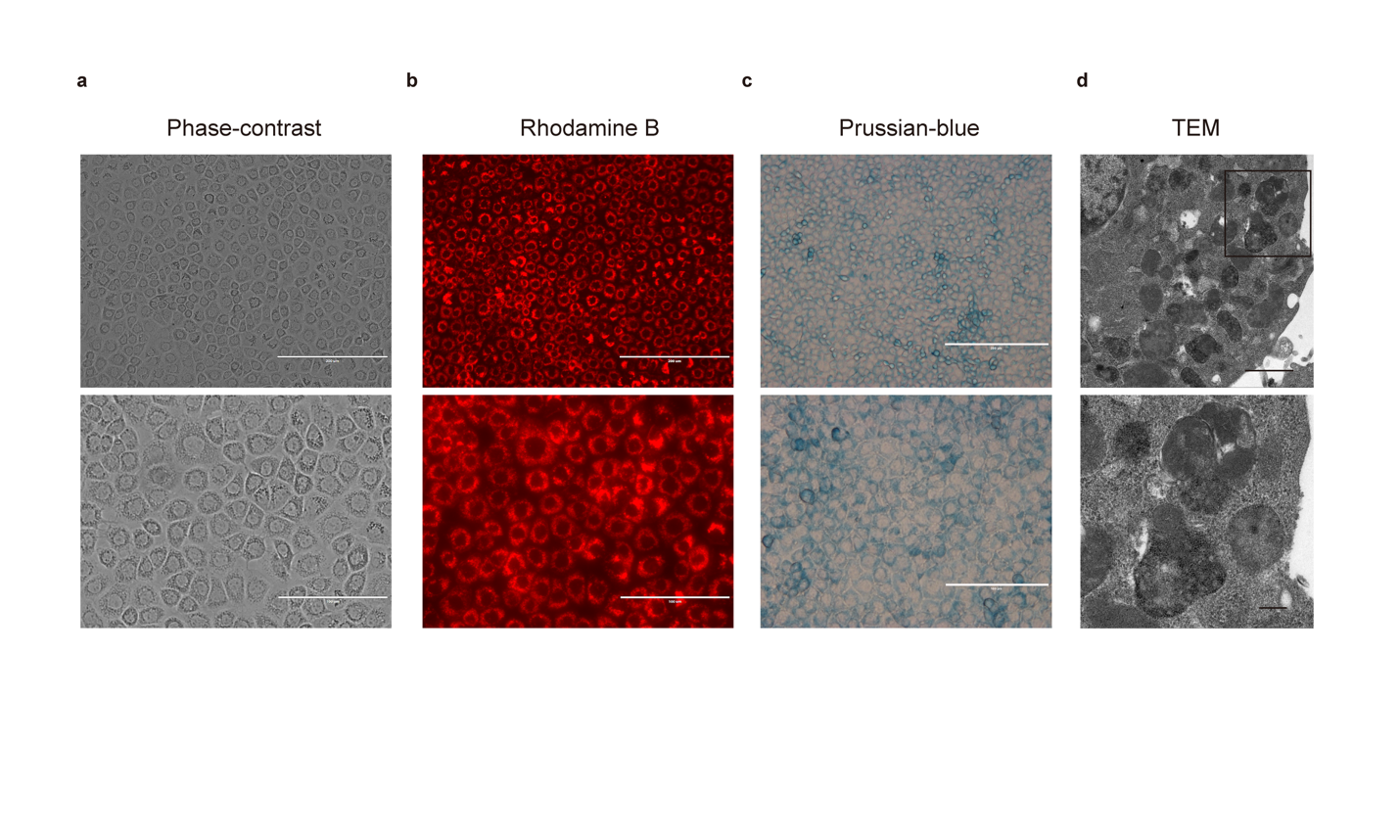
Figure. S9.

**Fig. S9** After USPIO incorporation into WB-F344 cells for 16 h, **a** Phase-contrast (scale bar, 200 μm and 100 μm), **b** Rhodamine B (scale bar, 200 μm and 100 μm) and **c** Prussian-blue (scale bar, 200 μm and 100 μm) staining images were captured, and the results indicated that over 90% of the cells incorporated USPIO (magnification, 200× and 400×). **d** The transmission electron microscopy (TEM) further demonstrated the presence of USPIO particles in the cytoplasm of WB-F344 cells (scale bar, 1 μm and 200 nm, magnification, 1700× and 14500×). Shown here are representative images from three experiments with similar results.


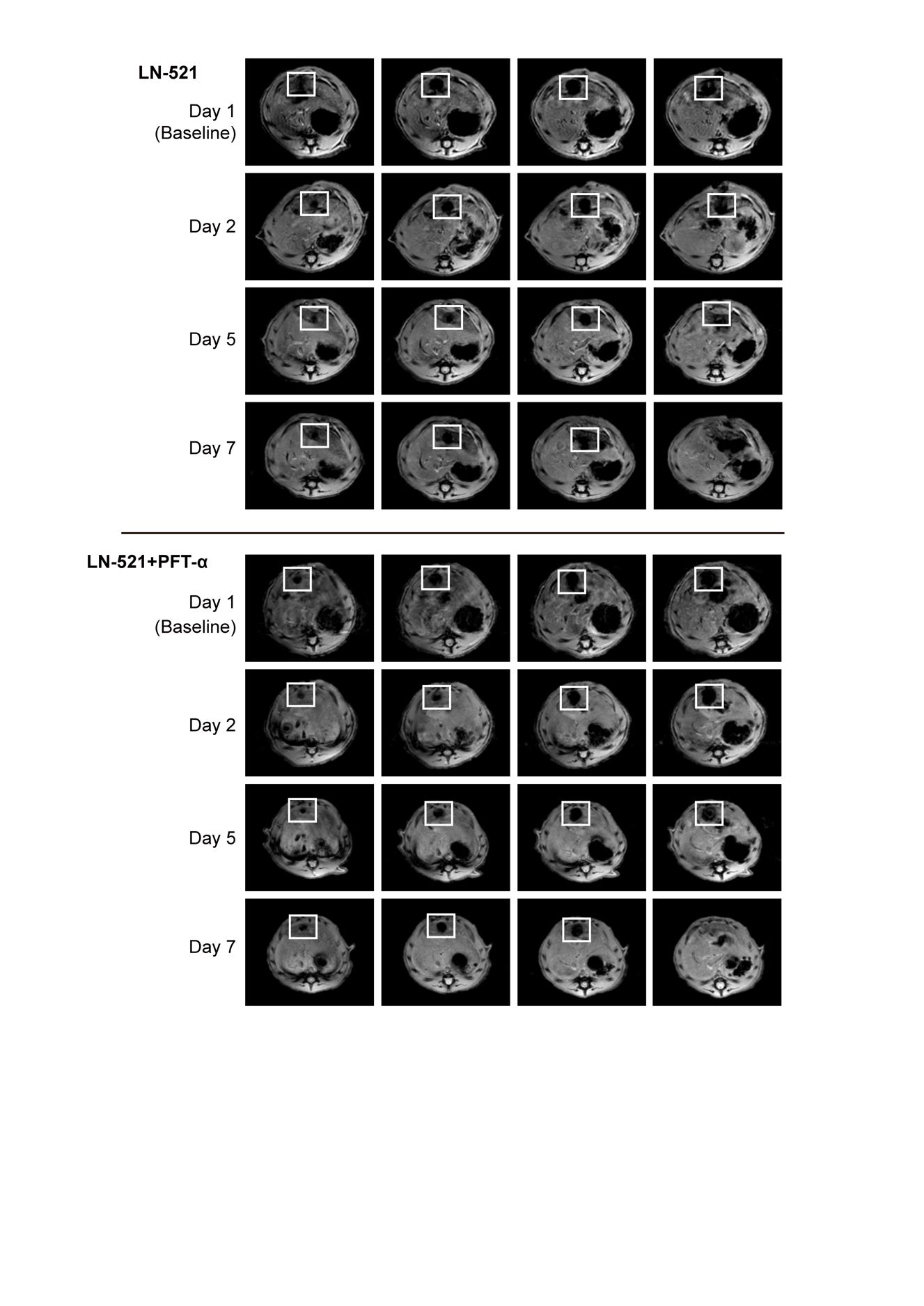
Figure. S10.

**Fig. S10** WB-F344 cells were transplanted into rat livers on the day of PH, and MR imaging of the rats receiving cells mixed with LN-521, with or without added PFT-α, was performed on the day of transplantation (Day 1, also the Baseline) and following 2, 5, and 7 days (n=6 per group). A representative set of T2*-weighted MR images of rats from each group are shown; the white frames indicate the hypointense areas consistent with injected cells.


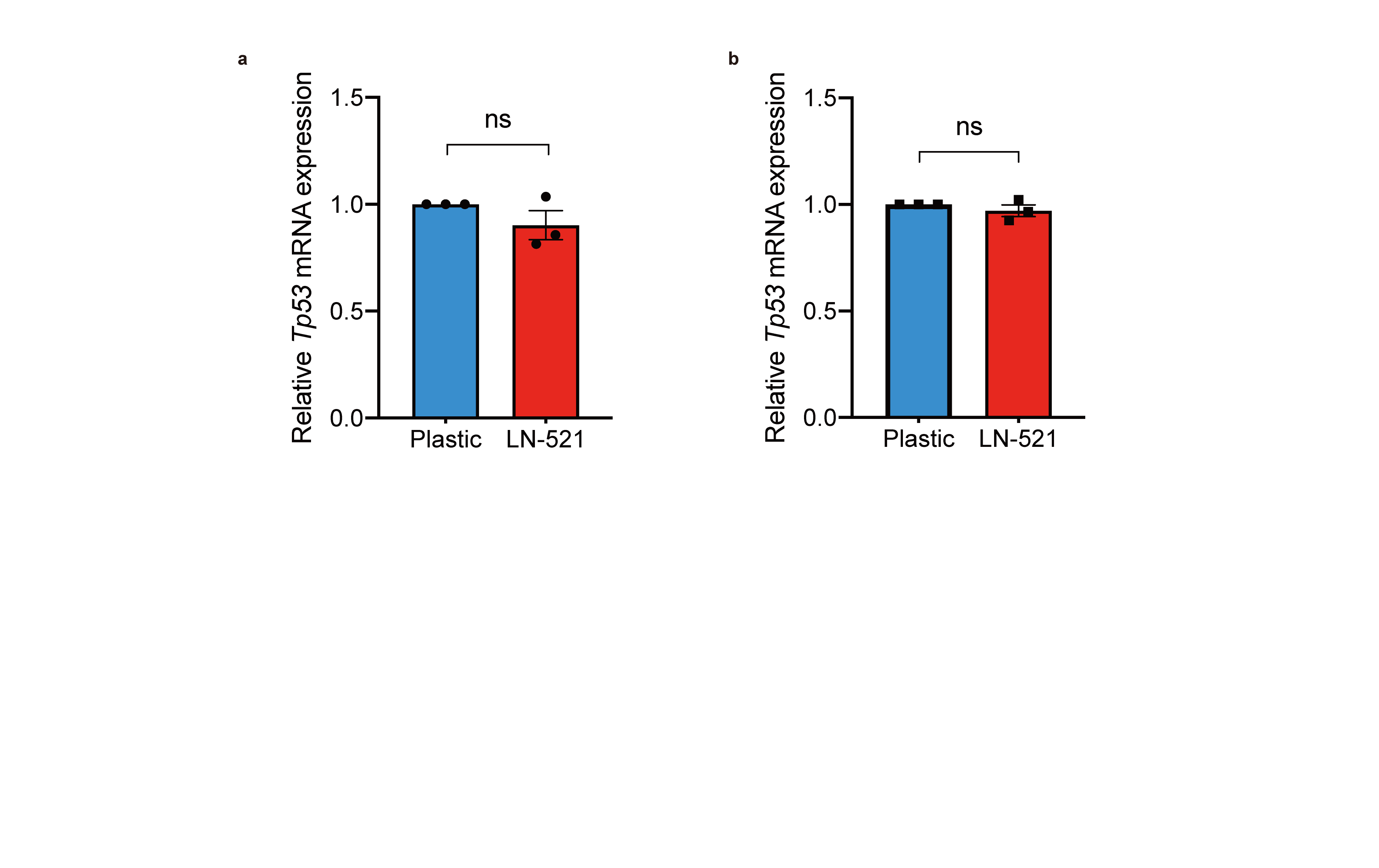
Figure. S11.

**Fig. S11** Transcriptional levels of *Tp53* analyzed by real-time qPCR in **a** WB-F344 cells and **b** Thy-1^+^ oval cells grown on plates coated with LN-521 or uncoated plastic plates. The graphs represent the quantification of mRNA levels of *Tp53* normalized to the control gene *Gapdh* with reference to the levels on the uncoated plastic plate. Experiments were performed in triplicate and data are expressed as means ± SEM. ns, not significant according to paired Student’s *t*-test.


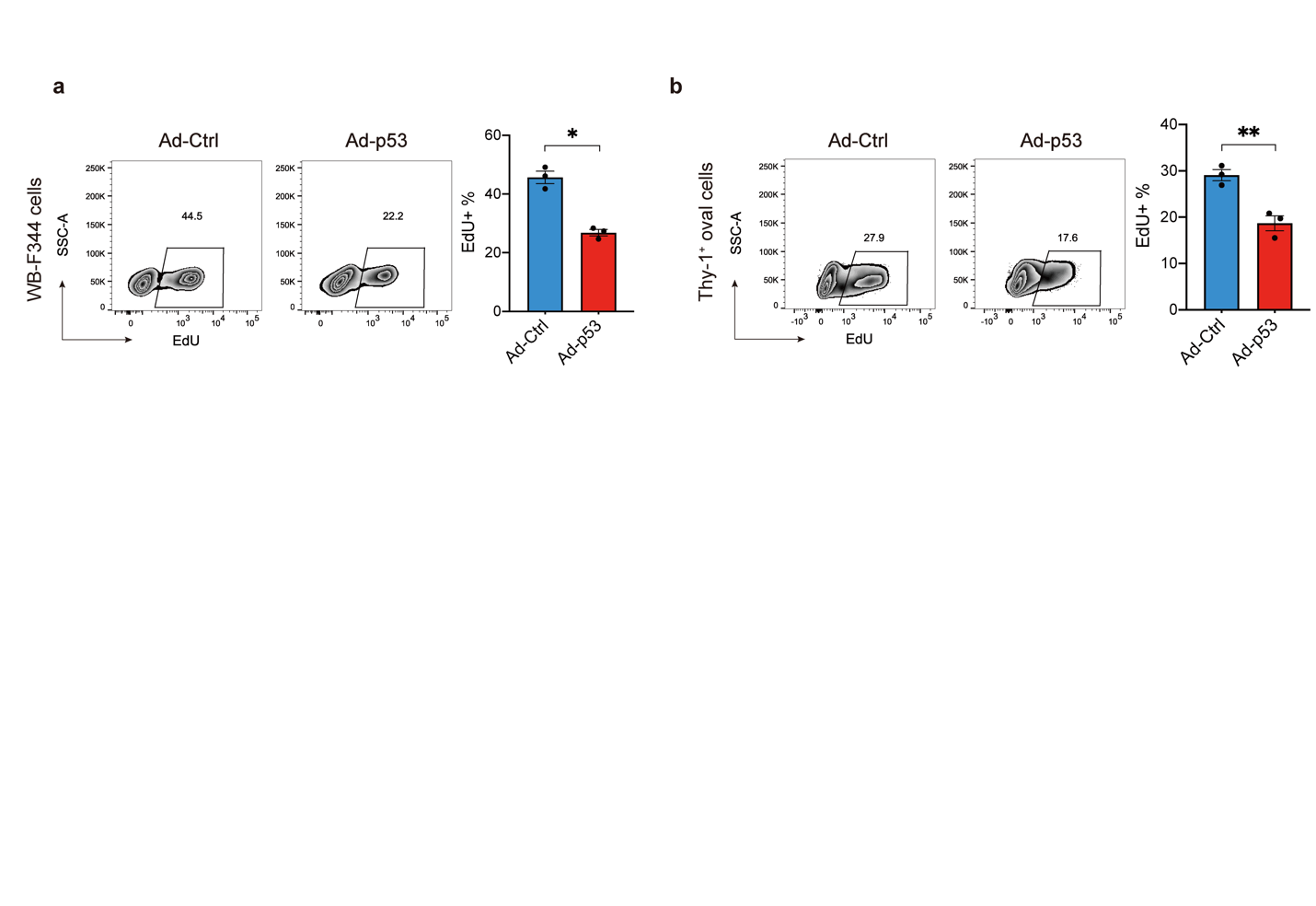
Figure. S12.

**Fig. S12 The proliferation of HPCs was inhibited by the ectopic overexpression of wild-type p53.** **a** WB-F344 cells and **b** Thy-1^+^ oval cells cultured in complete medium were infected with Ad-p53 or empty adenoviral vector (Ad-Ctrl), and the proliferation activity was analyzed using the EdU incorporation assay. Data are representatives of three independent experiments. Flow cytometry analysis of the percentage of EdU^+^ cells was expressed as means ± SEM. **P* < 0.05 and ***P* < 0.01 according to paired Student’s *t*-test.


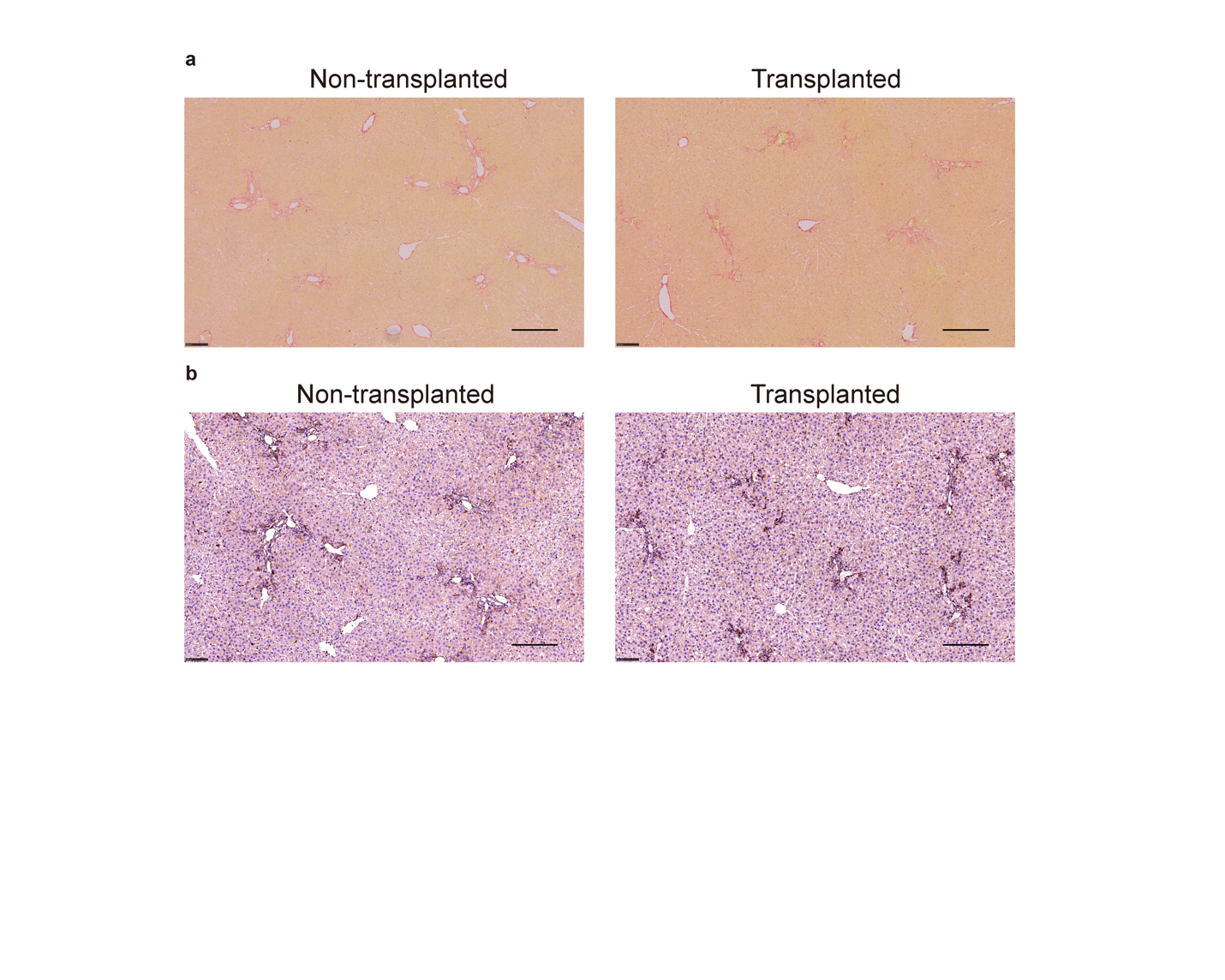
Figure. S13.

**Fig. S13 HPC transplantation had no effect on hepatic fibrogenesis.** Liver sections obtained from rats subjected to 2AAF/PH (Non-transplanted) and rats receiving 2AAF/PH in conjunction with cell transplantation (Transplanted) were stained with Sirius red or with a specific antibody against α-SMA to show collagen deposits. For cell transplantation, WB-F344 cells mixed with LN-521 were transplanted into rat livers on the day of PH. **a** Representative Sirius red stained sections of rat livers from the Non-transplanted and Transplanted groups. **b** Representative α-SMA immunostained sections of rat livers from the Non-transplanted and Transplanted groups. (scale bar, 200 μm, magnification, ×5 objective).


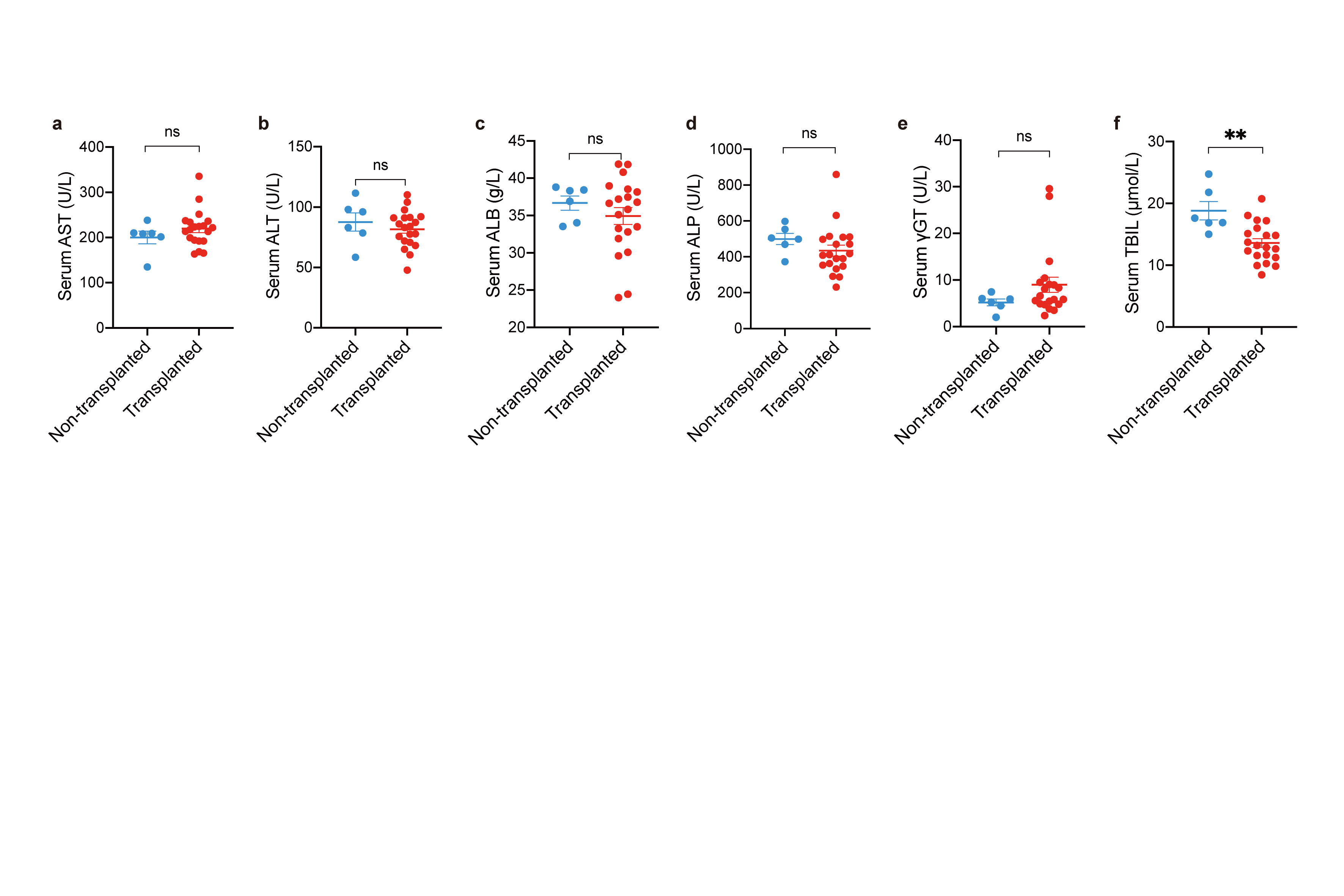
Figure. S14.

**Fig. S14** **HPC transplantation did not reduce liver injury.** Serum **a** aspartate aminotransferase (AST), **b** alkaline phosphatase (ALT), **c** albumin (ALB), **d** alkaline phosphatase (ALP), **e** gamma-glutamyl transpeptidase (γ-GT) and **f** total bilirubin (TBIL) levels of rats subjected to 2AAF/PH (Non-transplanted) and rats receiving 2AAF/PH in conjunction with cell transplantation (Transplanted) were compared to show the degree of liver injury. For cell transplantation, WB-F344 cells mixed with LN-521 were transplanted into rat livers on the day of PH. n=6 in Non-transplanted group and n=20 in Transplanted group. Data are represented as means ± SEM. ns, not significant and ***P* < 0.01 according to Student’s *t*- test.


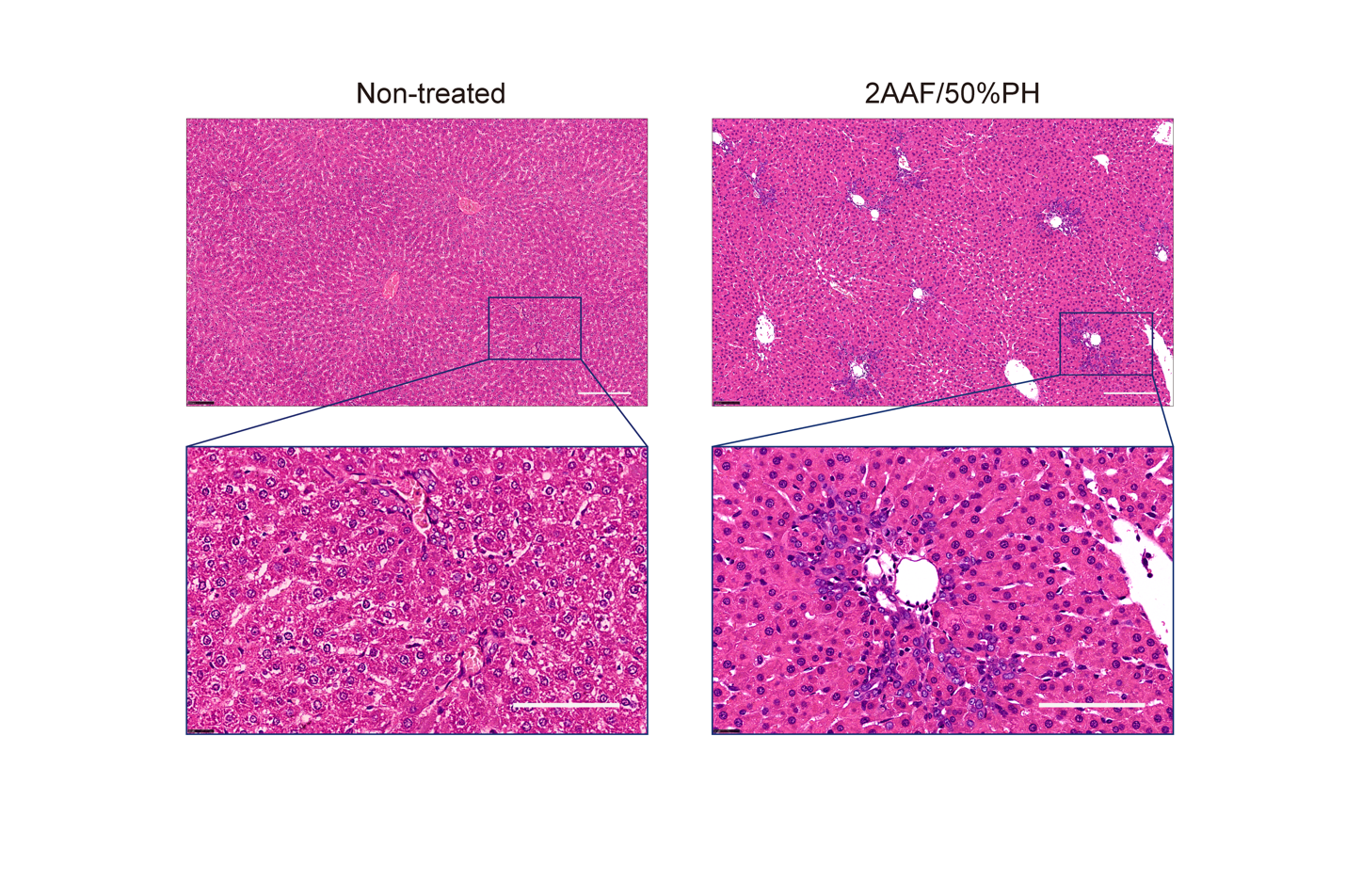
Figure. S15.

**Fig. S15 HPC activation in rats administrated 2-AAF in combination with 50% PH.** Representative hematoxylin and eosin staining of liver sections taken from rats seven days after PH (Right). Liver section from rats without treatment is the negative control (Left) (scale bar, 200 μm and100 μm, magnification, ×5 objective and ×20 objective).


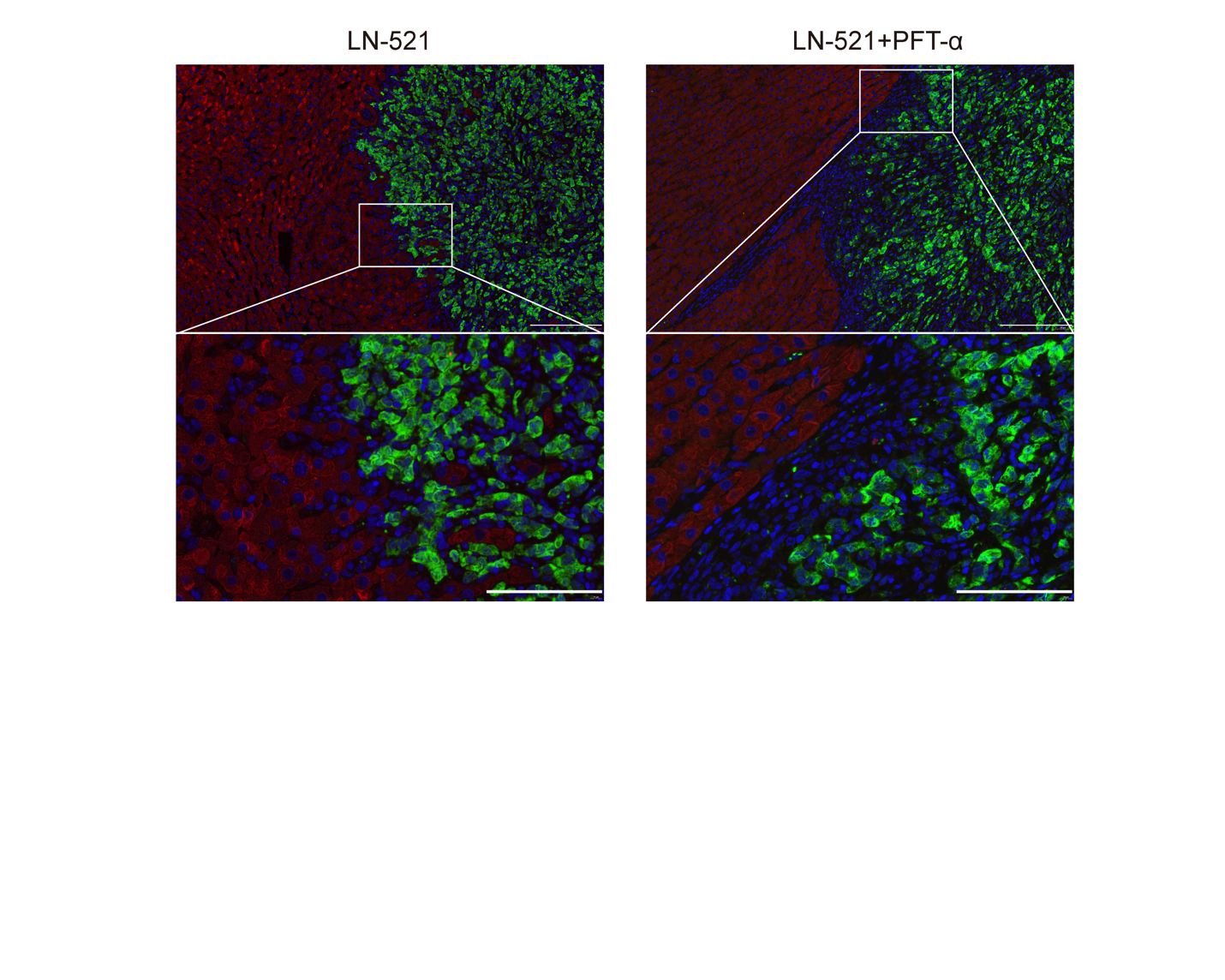
Figure. S16.

**Fig. S16 Determination of the retention and hepatic differentiation of transplanted cells within the graft site.** WB-F344 cells were transplanted into rat livers on the day of PH and immunofluorescence staining of the liver sections from rats receiving cells mixed with LN-521, with or without added PFT-α, was performed using luciferase-specific (green) and CYP2D6-specific (red) antibodies on day 12 upon transplantation. Large patches of luciferase-positive cells were observed in the inoculated liver, confirming the retention of transplanted cells. At the same time, the luciferase-expressing cells adjacent to host luciferase-negative hepatocytes did not co-express CYP2D6, which suggested that the transplanted cells did not differentiate into hepatocytes. Representative images are shown (scale bar, 200 μm and 100 μm, magnification, ×25 objective and ×80 objective).

Table S1. Primers for real-time qPCR detection

**Species: rats**

| **Gene** | **Primers (5’-3’) Forward** | **Primers (5’-3’) Reverse** |
| --- | --- | --- |
| *Laminin α1* | CGACTTAGCCAACGGGAAGT | CAATTCGGGAAACCCCGGTA |
| *Laminin α2* | CCCGGACCAACCTTAGGAAC | ACGGGCTGAAATTGTTGCTG |
| *Laminin α3* | AGTCCGTCTGCCAAATGACC | TTCACCCGGTCCATAACTGC |
| *Laminin α4* | CTGCATGTGTTCTACGACTTTGG | ATCGTTGATCTGGGCCTTTTT |
| *Laminin α5* | GACCACAGCGTGTGTTTGAC | CGGGAAACCTTCTGAGCTGT |
| *Laminin β1* | ATTGAACAATAGCTGCTCCG | ATGGTCCAGGGTGGTGAAGT |
| *Laminin β2* | GTACCCACACGGATGGAGTG | GAAGTAGGCCCAAGCGAAGT |
| *Laminin β3* | TTCTGCAGCCATTCGTCAGT | AAGTCACAGTCACAGGCTCG |
| *Laminin γ1* | GACCCCATCTAGTTCCTGCG | AGAAGCCCACTAGGAAACGC |
| *Laminin γ2* | CAAGCCTTGTTCATCTCATGTCA | GAACGCAGAGAAATGTGTCCAA |
| *Laminin γ3* | GCGGGTATACACAGAGCACTT | AGCCCAACATAACAGACCCC |
| *Tp53* | CCCCTGAAGACTGGATAACTGT | CAGGAGCTGACACTTGGAGG |
| *Cebpa* | GACCATCCGCCTTGTGTGTA | CTGACATTGCACAAGGCACC |
| *Ggt* | AAGGCTCCACTCACACCAAA | CGATTCTTCATGGCTCGTCCA |
| *Dlk1* | TTCTTTCCGCTGGACACCCG | GGTCGCACTCAGCCCCATA |
| *Sox9* | GTCGGTGAAGAATGGGCAAG | GACCCTGAGATTGCCCGGA |
| *Gapdh* | GGCACAGTCAAGGCTGAGAATG | ATGGTGGTGAAGACGCCAGTA |

Table S2. Antibodies application

| **Antibody** | **Cat NO., Manufacturer** | **Application** |
| --- | --- | --- |
| Laminin α2 | ab236762, Abcam | 1:1000 for WB |
| Laminin α4 | SC-16590, Santa | 1:1000 for WB |
| Laminin α5 | ab184330, Abcam | 1:1000 for WB |
| Laminin β1 | ab108536, Abcam | 1:1000 for WB |
| Laminin β2 | ab277521, Abcam | 1:1000 for WB |
| Laminin β3 | ab150385, Abcam | 1:1000 for WB |
| Laminin γ1 | SC-5584, Santa | 1:800 for WB |
| GAPDH | ab8245, Abcam | 1:5000 for WB |
| FAK | 3285, Cell Signaling Technology | 1:1000 for WB |
| p-FAK(Tyr397) | 3283, Cell Signaling Technology | 1:1000 for WB |
| Src | 2109, Cell Signaling Technology | 1:1000 for WB |
| p-Src(Tyr416) | 6943, Cell Signaling Technology | 1:1000 for WB |
| Paxillin | 2542, Cell Signaling Technology | 1:1000 for WB |
| p-Paxillin (Tyr118) | 2541, Cell Signaling Technology | 1:1000 for WB |
| Akt | 4691, Cell Signaling Technology | 1:1000 for WB |
| p-Akt (Ser473) | ab81283, Abcam | 1:2000 for WB |
| p-Rb | 8516, Cell Signaling Technology  ab184796, Abcam | 1:1000 for WB |
| CDK2 | sc-6248, Santa Cruz Biotechnology | 1:500 for WB |
| p-CDK2 | 2561, Santa Cruz Biotechnology | 1:1000 for WB |
| Cyclin D1 | ab134175, Abcam | 1:10000 for WB |
| Cyclin E | 20808, Cell Signaling Technology | 1:1000 for WB |
| p53 | 2524, Cell Signaling Technology | 1:1000 for WB |
| p27^Kip1^ | ab32034, Abcam | 1:5000 for WB |
| p21^Cip1^ | ab109199, Abcam | 1:1000 for WB |
| CDK4 | ab199728, Abcam | 1:2000 for WB  1:200 for IF |
| Luciferase | ab185924, Abcam | 1:500 for IF |
| CYP2D6 | 17868-1-AP | 1:200 for IF |
| α-SMA | ab7817, Abcam | 1:200 for IHC |
| β-actin | A01010-2, Abbkine Scientific | 1:5000 for WB |
| Flag-Tag | F-1804, Sigma | 1:2000 for WB |
| CD90 | 554894, BD Biosciences | recommended |
| Integrin-α1 | 555000, BD Biosciences | recommended |
| Integrin-α2 | 559987, BD Biosciences | recommended |
| Integrin-α3 | sc-7019, Santa Cruz Biotechnology | recommended |
| Integrin-α4 | 553348, BD Biosciences | recommended |
| Integrin-α5 | 103908, Biolegend | recommended |
| Integrin-α6 | sc-59920, Santa Cruz Biotechnology | recommended |
| Integrin-αv | ab124968, Abcam | recommended |
| Integrin-β1 | 102210, Biolegend | recommended |
| Integrin-β2 | 554976, BD Biosciences | recommended |
| Integrin-β3 | 104310, Biolegend | recommended |
| Integrin-β4 | 21738-1-AP, ProteinTech Group | recommended |
| Integrin-β5 | AB1926, Merck Millipore | recommended |
| Anti-Rabbit IgG | ab172730, Abcam | recommended |
| Anti-Hamster IgG | HTK888, Biolegend | recommended |
| Anti-Mouse IgG2a | 553454, BD Biosciences | recommended |
| Anti-Mouse IgG1 | 553447, BD Biosciences | recommended |
| Anti-Hamster IgG2 | 553961, BD Biosciences | recommended |
| Anti-Hamster IgM | 553957, BD Biosciences | recommended |

IF, Immunofluorescence; WB, Western blot; FACS, Fluorescence activated Cell Sorting; IHC, Immunohistochemistry

Table S3. Abbreviation list

| HPCs | hepatic progenitor cells |
| --- | --- |
| LN-521 | Laminin 521 |
| LN-111 | Laminin 111 |
| LN-211 | Laminin 211 |
| LN-332 | Laminin 332 |
| LN-411 | Laminin 411 |
| LN-421 | Laminin 421 |
| LN-511 | Laminin 511 |
| Lama1 | Laminin α1 |
| Lama2 | Laminin α2 |
| Lama3 | Laminin α3 |
| Lama4 | Laminin α4 |
| Lama5 | Laminin α5 |
| Lamb1 | Laminin β1 |
| Lamb2 | Laminin β2 |
| Lamb3 | Laminin β3 |
| Lamc1 | Laminin γ1 |
| Lamc2 | Laminin γ2 |
| Lamc3 | Laminin γ3 |
| ECM | extracellular matrix |
| PFT-α | Pifithrin-α |
| GAPDH | glyceraldehyde-3-phosphate dehydrogenase |
| MEFs | mouse embryonic fibroblasts |
| mESCs | murine embryonic stem cells |
| ADMSCs | adipose-derived mesenchymal stem cells |
| hESCs | human embryonic stem cells |
| hiPSCs | human induced pluripotent stem cells |
| hPSCs | human pluripotent stem cells |
| RPCs | retinal progenitor cells |
| NPCs | non-parenchymal cells |
| HSCs | hepatic stellate cells |
| CDK2 | cyclin-dependent kinases 2 |
| CDK4 | cyclin-dependent kinases 4 |
| CDKIs | cyclin-dependent kinase inhibitors |
| NLSs | nuclear localization signals |
| IDPs | intrinsically disordered proteins |
| PI3K | PI 3 Kinase |
| FAK | focal adhesion kinase |
| ILK | integrin-linked kinase |
| Rap1 | ras‐associated protein-1 |
| MAPK | mitogen-activated protein kinase |
| 2AAF/PH | 2- acetylaminofluorene/ partial hepatectomy |
| MRI | Magnetic Resonance Imaging |
| BLI | bioluminescence imaging |
| USPIO | ultrasmall superparamagnetic iron oxide |
| TEM | transmission electron microscopy |
| SNR | signal to noise ratio |
| ROI | regions of interest |
| SPSS | Statistical Product and Service Solutions |
| siRNA | small interfering RNA |
| TGF-α | transforming growth factor α |
| IGF I | insulin-like growth factor I |
| HGF | hepatocyte growth factor |
| EGF | epidermal growth factor |
| PAHs | polycyclic aromatic hydrocarbons |
| CDE | choline-deficient, ethionine-supplemented |
| AST | aspartate aminotransferase |
| ALT | alkaline phosphatase |
| ALB | albumin |
| ALP | alkaline phosphatase |
| γ-GT | gamma-glutamyl transpeptidase |
| TBIL | total bilirubin |
| IF | immunofluorescence |
| FCM | Flow Cytometry |
| WB | western blot |
| FACS | Fluorescence activated Cell Sorting |
| DAPI | 4',6-diamidino-2-phenylindole |
| OD | optical density |
| SPF | specific pathogen-free |
| FBS | fetal bovine serum |
| DMEM | Dulbecco’s Modified Eagle Medium |
| IMDM | Iscove's Modified Dulbecco's Medium |
| BSA | bovine serum albumin |
| PBS | phosphate buffer saline |
| FITC | fluorescein isothiocyanate |
| PCR | polymerase chain reaction |
| SDS | sodium dodecyl sulfate |
| PVDF | polyvinylidene fluoride |
